# Reconstitution of the lipid-linked oligosaccharide pathway for assembly of high-mannose *N-*glycans

**DOI:** 10.1038/s41467-019-09752-3

**Published:** 2019-04-18

**Authors:** Sheng-Tao Li, Tian-Tian Lu, Xin-Xin Xu, Yi Ding, Zijie Li, Toshihiko Kitajima, Neta Dean, Ning Wang, Xiao-Dong Gao

**Affiliations:** 1 Key Laboratory of Carbohydrate Chemistry and Biotechnology, Ministry of Education, School of Biotechnology, Jiangnan University, 214122 Wuxi, China; 2 Department of Biochemistry and Cell Biology, Stony Brook University, Stony Brook, NY 11794-5215 USA

**Keywords:** Biocatalysis, Carbohydrates, Transferases, Glycobiology

## Abstract

The asparagine (N)-linked Man9GlcNAc2 is required for glycoprotein folding and secretion. Understanding how its structure contributes to these functions has been stymied by our inability to produce this glycan as a homogenous structure of sufficient quantities for study. Here, we report the high yield chemoenzymatic synthesis of Man9GlcNAc2 and its biosynthetic intermediates by reconstituting the eukaryotic lipid-linked oligosaccharide (LLO) pathway. Endoplasmic reticulum mannosyltransferases (MTases) are expressed in *E. coli* and used for mannosylation of the dolichol mimic, phytanyl pyrophosphate GlcNAc2. These recombinant MTases recognize unique substrates and when combined, synthesize end products that precisely mimic those in vivo, demonstrating that ordered assembly of LLO is due to the strict enzyme substrate specificity. Indeed, non-physiological glycans are produced only when the luminal MTases are challenged with cytosolic substrates. Reconstitution of the LLO pathway to synthesize Man9GlcNAc2 in vitro provides an important tool for functional studies of the N-linked glycoprotein biosynthesis pathway.

## Abstract (web summary)

Attachment of the oligosaccharide Man9GlcNAc2 is required for glycoprotein folding and secretion but synthesizing this compound for structural and functional studies has remained challenging. Here, the authors achieve efficient Man9GlcNAc2 synthesis by reconstituting its biosynthetic pathway in vitro.

## Introduction

N-linked glycosylation is an essential modification that regulates protein structure and function^1,2^. The N-linked glycan is processed very differently in species-specific, tissue-specific, and cell-specific ways, leading to an immensely complex glycome. Despite their heterogeneity, most of N-glycans share a common Glc3Man9GlcNAc2 precursor oligosaccharide that is pre-assembled on the endoplasmic reticulum (ER) membrane before it is transferred to protein. Fourteen mono-saccharides are sequentially added onto a dolichyl pyrophosphate (PP-Dol) membrane anchor by ER membrane-associated Alg (asparagine-linked glycosylation) glycosyltransferases (GTases)^3^. Once assembled, the Glc3Man9GlcNAc2 oligosaccharide is transferred to the target protein by oligosaccharyltransferase (OST), which catalyzes the formation of an N-glycosidic bond to an asparagine within the Asn-X-(Ser/Thr) consensus sequence^3,4^. After its transfer, Glc3Man9GlcNAc2 is modified by removal or re-addition of glucoses under the regulation of the calnexin–calreticulin cycle. Production of deglucosylated protein-bound Man9GlcNAc2 (M9GN2) is the signal that tells the cell a glycoprotein has acquired its native conformation^5,6^, and hence is competent to exit the ER for further cell-type-specific glycosylation in the Golgi. Errors in the synthesis, transfer, or modification of the N-linked glycan causes glycoproteins to be recognized by quality control systems, preventing their exit from the ER and targeting them for degradation^7–9^. Its critical position at the junction of glycoprotein folding, quality control, and transport from the ER underscores the importance of understanding the molecular details of M9GN2 for a range of biological and pharmacological studies, including glycan arrays^10,11^, vaccine production^12,13^, and glycoprotein quality control^14,15^.

**Fig. 1:**
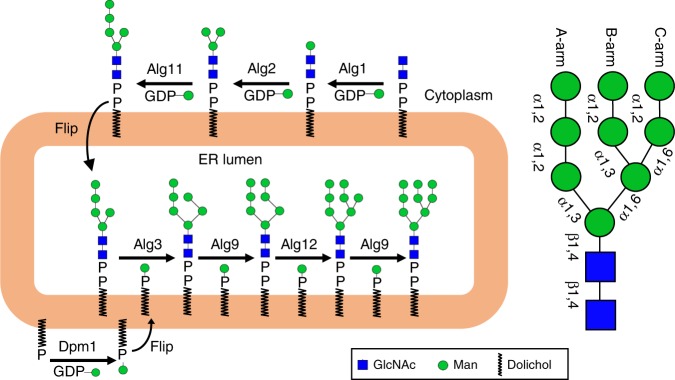
Mannosylation in the eukaryotic LLO biosynthesis pathway. Alg mannosyltransferases (MTases) catalyze formation of M9GN2-PP-Dol by sequentially adding mannoses to GN2-PP-Dol. Cytosolic reactions use the nucleotide sugar GDP-Man donor, while luminal enzymes use Man-P-Dol, synthesized by Dpm1. Linkages of each sugar are indicated on the right, as are the A, B and C arms of the triantennary oligosaccharide

In eukaryotic cells, stepwise assembly of M9GN2 occurs on the ER membrane in two topologically distinct set of reactions (Fig. 1)^3^. First, on the cytosolic face, the Alg7/Alg13/Alg14 complex adds two N-acetylglucosamines from uridine diphosphate N-acetylglucosamine (UDP-GlcNAc) to make dolichyl pyrophosphate GlcNAc2 (GN2-PP-Dol)^16–21^. The Alg1, Alg2 and Alg11 mannosyltransferases (MTases) then add five mannoses from guanosine diphosphate mannose (GDP-Man) to make Man5GlcNAc2 (M5GN2-PP-Dol)^22–26^. Second, after M5GN2-PP-Dol is flipped from the cytosolic face of the ER into the lumen, four additional mannoses are added by the Alg3, Alg9, and Alg12 MTases to form M9GN2-PP-Dol (Fig. 1)^27–30^. In contrast to the cytosolic mannosylations of M5GN2 that use GDP-Man as sugar donor, the luminal mannosylations use dolichyl phosphate mannose, whose synthesis is catalyzed by dolichol phosphate mannose synthase (Dpm1, Fig. 1)^31^. Thus, biosynthesis of M9GN2-PP-Dol requires expression of nine different Alg GTases and three different donor sugar substrates.

Much effort has been devoted to the production of structurally homogenous M9GN2 substrate in amounts sufficient for functional and structural studies^32–34^. Isolation of M9GN2 from natural sources (i.e. egg yolk and soybean) is limited by low recovery^35,36^. Chemical synthesis of various high-mannose N-glycans has also been accomplished, resulting in higher yields but is labor-intensive and time-consuming^37–40^. Chemoenzymatic synthesis using lipid-linked oligosaccharide (LLO) substrates with simplified lipids has proved useful for producing some M9GN2 precursors, including M3GN2 and M5GN2^25,41–44^. However, full length lipid-linked M9GN2 production has thus far been limited by its enzymatic complexity, which requires purification of a lipid carrier, various sugar donors, and all the Alg MTases.

Here, we overcome these challenges and describe efficient production of full length M9GN2 oligosaccharide in vitro using recombinant Alg proteins expressed from *E. coli*. Reconstitution of the entire LLO pathway from GlcNAc2 to M9GN2 is achieved in two successive one-pot reactions, which correspond to the reactions that occur on the cytosolic and on the luminal faces of the ER in vivo.

## Results

### Chemo-enzymatic synthesis of M5GN2

**Fig. 2:**
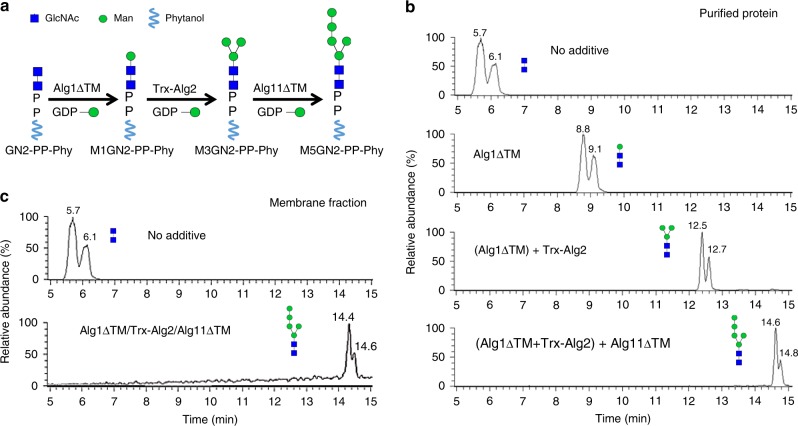
In vitro M5GN2-PP-Phy synthesis. **a** Schematic diagram of sequential mannosylation reactions catalyzed by Alg1ΔTM, Trx-Alg2, and Alg11ΔTM. **b** UPLC chromatograms of hydrolyzed glycans from reactions with various combinations of purified MTases. Each segment of sequential reactions was incubated for 12 h, as described in Methods. Reactions that included GN2-PP-Phy, GDP-Man, and Alg1ΔTM produced M1GN2 (Alg1ΔTM); sequential addition of Alg1ΔTM and Trx-Alg2 produced M3GN2 [(Alg1ΔTM)+Trx-Alg2]; sequential addition of Alg1ΔTM, Trx-Alg2, and Alg11ΔTM generated M5GN2 [(Alg1ΔTM+Trx-Alg2)+Alg11ΔTM]. **c** UPLC chromatogram of hydrolyzed glycans from a one-pot reaction containing GN2-PP-Phy, GDP-Man, and a membrane fraction purified from *E. coli* that co-expressed Alg1ΔTM, Trx-Alg2, and Alg11ΔTM

We previously reported efficient synthesis of the M3GN2 intermediate oligosaccharide using recombinant Alg1 and Alg2 MTases, and a synthetic phytanyl pyrophosphate GlcNAc2 (GN2-PP-Phy) substrate^25,45^. In those studies, M3GN2-PP-Phy was sequentially synthesized with recombinant His-tagged *S. cerevisiae* Alg1 lacking its transmembrane domain (TMD) (Alg1ΔTM) to produce M1GN2, and full length Alg2, including its TMD and an N-terminal thioredoxin tag (Trx-Alg2) to produce M3GN2. The Alg2 reaction was performed in the presence of *E. coli* membrane fraction^25,45^. To produce M5GN2-PP-Phy, we purified Alg11, which adds the next two mannoses on M3GN2-PP-Phy. *S. cerevisiae* Alg11 lacking its N-terminal TMD was overexpressed and purified from *E. coli* (Supplementary Fig. 1a and b). GN2-PP-Phy and GDP-Man were incubated sequentially with recombinant Alg1ΔTM, Trx-Alg2, and Alg11ΔTM, to produce M5GN2-PP-Phy (Fig. 2a). The reactions were quenched by addition of acid to release PP-Phy from the oligosaccharides, which were purified and analyzed by ultra-performance liquid chromatography–mass spectroscopy (UPLC–MS). Without added MTase, acid hydrolyzed GlcNAc2 (GN2) eluted in two peaks (5.7 and 6.1 min, Fig. 2b) designated the alpha and beta anomeric isomers of GN2^45^. Addition of different combinations of Alg1∆TM, Alg2, and Alg11∆TM resulted in a loss of GN2 and a shift in the UPLC retention time of higher molecular weight glycan products (detected by MS) (Supplementary Fig. 2). In the sequential reactions, the first mannose was added by Alg1ΔTM to produce the trisaccharide M1GN2; addition of Trx-Alg2 with membrane fraction of *E. coli* (since the bilayer formation is critical for Alg2 in vitro activity^25^, membranes are always included with Trx-Alg2) to the first reaction extended M1GN2 with two additional mannoses, leading to M3GN2; final addition of Alg11ΔTM produced M5GN2, which eluted in two peaks that originated from the target product, with retention times at about 14 min in UPLC (Fig. 2b).

In vivo, Alg1, Alg2, and Alg11 form a multimeric MTase complex on the cytosolic face of the ER membrane^42^. Assembly of this complex is required for catalysis of the five sequential mannosylations that lead to M5GN2^46,47^. In an attempt to simplify enzymatic M5GN2 production, we tested if co-expression of recombinant Alg1ΔTM, Trx-Alg2, and Alg11ΔTM could form an active complex in purified *E. coli* membranes. If so, membrane fractions from *E. coli* containing this complex would enable one-pot mannosylation of GN2-PP-Phy to produce M5GN2-PP-Phy. Membrane fractions isolated from *E*. coli expressing these enzymes and lysed by sonication were analyzed by western blotting. The result demonstrated that when co-expressed, Alg1ΔTM, Trx-Alg2, and Alg11ΔTM in membranes prepared from *E. coli* (see Methods) migrated with a molecular weight indistinguishable from the corresponding proteins purified individually (Supplementary Fig. 1c). This Alg1ΔTM, Trx-Alg2, and Alg11ΔTM-containing membrane fraction was incubated with GN2-PP-Phy and GDP-Man. After 12 h, the reaction was quenched and acid-released oligosaccharides were analyzed by UPLC–MS. The result of this experiment suggested that M5GN2 could indeed be produced in a one-pot reaction since GN2-PP-Phy was completely converted to M5GN2 (Fig. 2c), with a product yield of ~8 μg from 100 μL reaction volume.

All oligosaccharides generated by above reactions were confirmed by mass spectral analysis (Supplementary Fig. 2). To further determine the mannoside linkages of these M5GN2 glycans, we performed the treatment with specific mannosidases (Supplementary Fig. 3). In vivo, Alg1 attaches the first mannose to GN2-PP-Dol via an β1,4 linkage; Alg2 adds the second mannose via an α1,3 linkage and the third mannose via an α1,6 linkage (Fig. 1); Alg11 adds the fourth and fifth mannoses via an α1,2 linkage on the A-arm. If each of the mannoses in M5GN2 generated by above reactions is linked in the way they are in vivo, treatment with α1,2 mannosidase removes two α1,2 mannoses modified by Alg11ΔTM^43^; treatment with α1,2-3 mannosidase removes two α1,2 mannoses modified by Alg11ΔTM and one α1,3 mannose modified by Trx-Alg2; treatment with α1,2-3-mannosidase and α1,6 mannosidase will remove two α1,2 mannoses modified by Alg11ΔTM, one α1,3 mannose and one α1,6 mannose modified by Trx-Alg2^25^. It should be noticed that, in our study, the usual structures of LLOs are abbreviated as MxGN2, in which *x* indicates the number of Man residues. On the other hand, the nomenclature for those unusual and digested LLO intermediates, which will appear in the late part of the manuscript, be abbreviated as M*x*(A*y*/B*y*/C*y*)GN2, in which A/B/C indicates the A-arm, B-arm, or C-arm, respectively; *y* is the number for denoting Man residues disconnected from an particular arm of the M9GN2. All these structures of LLOs discussed in our study and their abbreviations have been summarized in Supplementary Table 1.

As shown in Supplementary Fig. 3, digestion of M5GN2 with α1,2-mannosidase yielded M3GN2 as predicted for its removal of two α1,2 mannoses from the A-arm; α1,2-3-mannosidase digestion removed two α1,2-linked mannoses and one additional α1,3-linked mannose to give M2AGN2; treating with α1,2-3-mannosidase and α1,6 mannosidase generated M1GN2. These structural analyses demonstrated the M5GN2-PP-Phy synthesized in vitro has the same glycan structure as that synthesized in vivo by the LLO pathway. A scale-up of one-pot reaction produced milligram quantities of the product, whose structure was also verified by mass analysis and NMR spectrum (see in Supplementary Notes 1–3, Supplementary Figs. 4a and 5a). Therefore, membrane fractions from *E. coli* that co-express yeast Alg1, Alg2, and Alg11 proteins provide a simple, inexpensive source of enzymes for chemoenzymatic synthesis of M5GN2 with 100% conversion rate.

### Synthesis of M9GN2 and its intermediates from M5GN2-PP-Phy

In vivo, after its synthesis on the cytosolic face of the ER, M5GN2-PP-Dol is translocated and extended to M9GN2 by the luminal Alg3, Alg9, and Alg12 MTases (Fig. 1)^3^. Alg3 attaches the sixth mannose to M5GN2-PP-Dol via an α1,3 linkage on the B-arm (Fig. 1)^27,30^; Alg9 adds the seventh mannose via an α1,2 linkage on the B-arm (Fig. 1)^28^; Alg12 adds the eighth mannose via an α1,6 linkage on the C-arm^29,48^; Alg9 also adds the ninth mannose via an α1,2 linkage on the C-arm (Fig. 1)^28^.

**Fig. 3:**
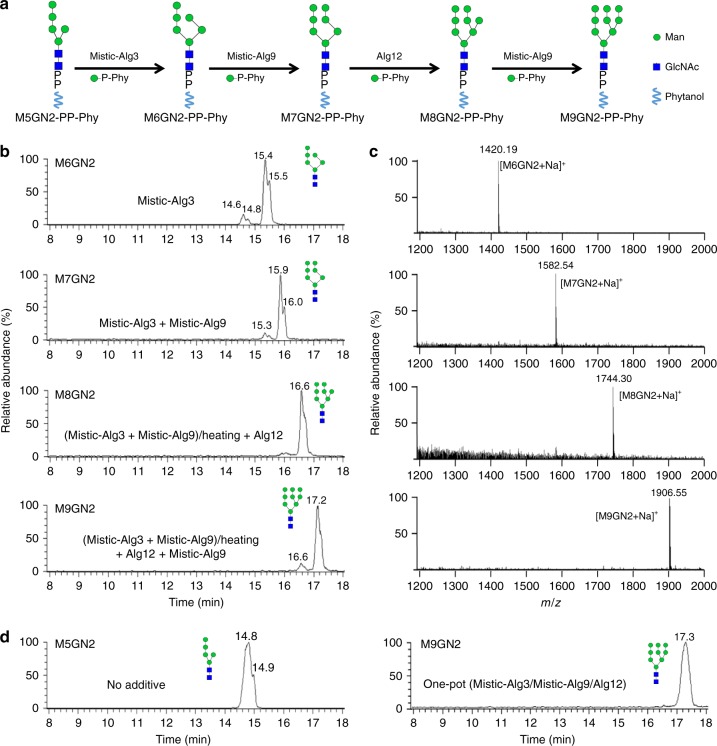
In vitro assembly of the M9GN2-PP-Phy. **a** Schematic diagram of sequential mannosylation reactions catalyzed by Mistic-Alg3, Mistic-Alg9, and Alg12. **b** UPLC chromatograms of hydrolyzed glycans from reactions with various combinations of membrane fraction purified from *E. coli* expressing either Alg3, Alg9, or Alg12. In the presence of M5GN2-PP-Phy and Man-P-Phy, addition of Mistic-Alg3 produced M6GN2 (Mistic-Alg3); sequential addition of Mistic-Alg3 and Mistic-Alg9 produced M7GN2 (Mistic-Alg3+Mistic-Alg9); the reaction with Mistic-Alg3 and Mistic-Alg9 was stopped by heating, then adding Alg12 produced M8GN2 [(Mistic-Alg3+Mistic-Alg9)/heat+Alg12]; sequential addition of Mistic-Alg3 for 12 h, Mistic-Alg9 for 12 h, Alg12 for 12 h, and Mistic-Alg9 for 12 h generated M9GN2 (Mistic-Alg3+Mistic-Alg9+Alg12). **c** Mass spectra of glycans released from Phy-PP-linked oligosaccharide products. Mass analyses showed the peaks eluted (**b**) at ~15.5, ~16.0, ~16.6, and ~17.2 min correspond to M6GN2 ([M6GN2+Na]^+^), M7GN2 ([M7GN2+Na]^+^), M8GN2 ([M8GN2+Na]^+^), and M9GN2 ([M9GN2+Na]^+^), respectively. **d** UPLC chromatogram of hydrolyzed glycans from reactions in which Mistic-Alg3, Mistic-Alg9, and Alg12 were added simultaneously for 20 h in one pot to generate M9GN2 from M5GN2-PP-Phy and Man-P-Phy

To perform the M5GN2 to M9GN2 extension reactions in vitro (Fig. 3a), recombinant yeast Alg3, Alg9, and Alg12 were purified from *E. coli*. All these enzymes contain multiple TMDs (TMHMM Server v. 2.0), which suggested that their expression in bacteria could be problematic. Indeed, attempts to express N-terminally His-tagged proteins were successful only for Alg12; both Alg3 and Alg9 were unstable in *E. coli*. Protein stability was improved dramatically by attaching a Mistic-tag to the N-terminus of Alg3 and Alg9. Mistic is a short bacterial protein used to enhance expression of integral membrane proteins in *E. coli*^49^. Expression of Mistic-Alg3, Mistic-Alg9 as well as Alg12 was confirmed by western blotting (Supplementary Fig. 1d). Since detergent extraction of these enzymes from *E. coli* membrane led to decrease of activity, mannosylation reactions to extend M5GN2 to M9GN2 were performed with membrane fractions from *E. coli* expressing recombinant Alg3, Alg9, and Alg12.

Since the nucleotide sugar donor GDP-Man cannot cross the ER membrane, luminal ER MTases, Alg3, Alg9, and Alg12 utilize lipid-linked Man-P-Dol as sugar donor (Fig. 1). Purification of Man-P-Dol is laborious, therefore we tested the feasibility of using another sugar donor more amenable to in vitro mannosylation. Since MTases recognize and extend phytanyl-linked glycan as efficiently as dolichol-linked glycan substrates^50,51^, we reasoned that recombinant Alg3, Alg9, and Alg12 might be able to use phytanyl-linked instead of dolichol-linked mannose (Man-P-Phy) as a sugar donor. Man-P-Phy is soluble so if this idea were correct, preparation of sugar donor for these in vitro reactions would be simplified. To test this idea, recombinant *S. cerevisiae* Dpm1^31^, which catalyzes addition of mannose from GDP-Man to dolichyl phosphate (P-Dol), was purified from *E. coli* and used to synthesize Man-P-Phy (Supplementary Fig. 6a and b). Purified Dpm1 was incubated with GDP-Man and phytanyl phosphate (P-Phy), and Man-P-Phy production was monitored by thin layer chromatography (TLC). As shown in Supplementary Fig. 6c, almost quantitative conversion of Man-P-Phy from P-Phy was observed, demonstrating efficient recognition of P-Phy by Dpm1. Man-P-Phy prepared by Dpm1 was used as sugar donor for extension of M5GN2 to M9GN2 by recombinant Alg3, Alg9, and Alg12.

**Fig. 4:**
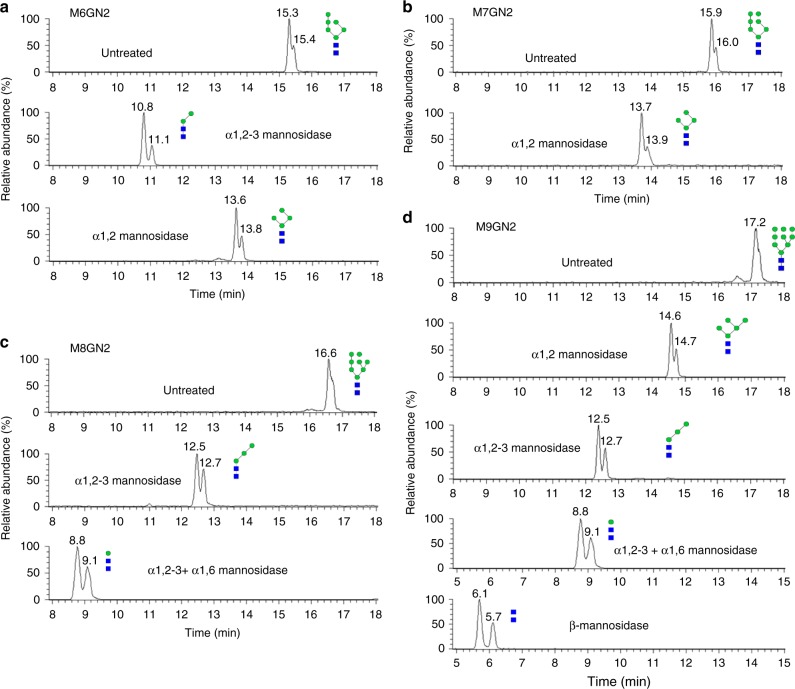
UPLC–MS analyses of mannosidase digestion of M9GN2 and precursors. Each of the glycans generated in experiments shown in Fig. 3b were digested with linkage-specific mannosidases, including: α1,2-mannosidase, which removes terminal α1,2 mannoses; α1,2-3-mannosidase, which removes terminal α1,2 mannoses and terminal α1,3 mannoses; α1,6-mannosidase, which removes terminal non-branched α1,6 mannoses; β-mannosidase, which removes terminal β-mannoses. Digestion products and their deduced structure are depicted schematically. **a** Digestion of M6GN2 with α1,2-3-mannosidase produced M2AGN2; while its digestion with α1,2-mannosidase produced M4A2BC2GN2. **b** Digestion of M7GN2 with α1,2-mannosidase produced M4A2BC2GN2. **c** Digestion of M8GN2 with α1,2-3-mannosidase produced M3A3B2CGN2; while its digestion with α1,2-3-mannosidase and α1,6-mannosidase produced M1GN2. **d** Digestion of M9GN2 with α1,2-mannosidase produced M5BCGN2; digestion with α1,2–3-mannosidase produced M3A3B2CGN2; digestion with both α1,2-3-mannosidase and α1,6-mannosidase produced M1GN2; further treatment with β-mannosidase produced GN2

To produce M6GN2-PP-Phy, M5GN2-PP-Phy was incubated with in situ prepared Man-P-Phy and a membrane fraction purified from *E. coli* expressing Mistic-Alg3. After 12 h, acid-hydrolyzed glycan products were analyzed by UPLC–MS. These glycan products eluted in a peak at ~15.4 min with a mass *m*/*z*: of 1420.19 ([M6GN2+Na]^+^), which corresponds to the predicted molecular weight of M6GN2 (Fig. 3b, c). To determine the mannoside linkage in M6GN2, it was subjected to treatment with specific mannosidases. In vivo, Alg3 attaches one mannose to M5GN2 via an α1,3 linkage. As shown in Fig. 4a, after digesting the product with α1,2-3-mannosidase, peaks corresponding to M2AGN2 were observed, suggesting two α1,2-linked mannoses and two α1,3-linked mannoses were removed; treatment with α1,2-mannosidase resulted in M4A2BC2GN2, indicating the removal of two α1,2-linked mannoses. These results demonstrated that Alg3-catalyzed mannose on M6GN2 bears the predicted α1,3-mannoside linkage. To gain information about the kinetics of this reaction, time-dependent conversion ratios were calculated by measuring the production of M6GN2 at different time points. After 4 h of incubation, the conversion ratio of reaction reached over 90%; by 12 h it had reached ~100% (Supplementary Fig. 7a). Thus, we chose 12 h incubation for performing the complete extension of M6GN2-PP-Phy from M5GN2-PP-Phy (Fig. 3b).

M7GN2-PP-Phy was produced by sequential addition of two mannoses to M5GN2-PP-Phy by Alg3 and Alg9. M5GN2-PP-Phy was incubated with membrane fractions from *E. coli* expressing Mistic-Alg3 for 12 h, after which Mistic-Alg9 added for an additional 12 h. The molecular weight and anomeric structure of the glycan products were verified by UPLC–MS and mannosidase digestion. Peaks eluting at ~15.9 min in UPLC possessed the mass peak *m*/*z*: 1582.54 ([M7GN2+Na]^+^), which correspond to M7GN2 (Fig. 3b, c). After treatment with α1,2-mannosidase, peaks corresponding to M4A2BC2GN2 were observed (Fig. 4b), indicating the removal of three α1,2-linked mannoses. These data demonstrated that the seventh Alg9-catalyzed mannose in M7GN2 is attached to M6GN2 by an α1,2-Man linkage. The 12 h incubation time used for Mistic-Alg9 extension in Fig. 3b was determined on a kinetic analysis of time-dependent conversion ratios (Supplementary Fig. 7b), which revealed nearly 100% conversion from M6GN2 to M7GN2 after 12 h.

M8GN2-PP-Phy was produced by addition of one mannose to M7GN2-PP-Phy by Alg12. After producing M7GN2-PP-Phy with Mistic-Alg3 and Mistic-Alg9, the reaction was stopped by heating at 100 °C to inactivate Alg3 and Alg9, cooled and then incubated with a membrane fraction from *E. coli* producing Alg12 for 12 h. Glycans produced in this reaction are eluted in UPLC–MS peaks with *m*/*z*: 1744.30, which corresponds to the predicted mass of M8GN2 (Fig. 3b, c). To confirm the newly formed Alg12-catalyzed Man–Man linkage in M8GN2 is identical to the in vivo α1,6-linked man, the product was subjected to mannosidase treatment. As shown in Fig. 4c, digestion of the product with α1,2-3-mannosidase removed two α1,2-linked mannoses from the A-arm, one α1,2-linked mannose from the B-arm, and two additional α1,3-linked mannoses (from A-arm and B-arm, respectively) to generate M3A3B2CGN2 with two α1,6 mannoside linkages on C arm. Further treatment with α1,6 mannosidase removed both these mannoses (Fig. 4c). These results demonstrated that the eighth mannose added by Alg12 is attached via the predicted α1,6-linkage. Kinetic analysis of recombinant Alg12 demonstrated it could convert about 80% of M7GN2-PP-Phy to M8GN2-PP-Phy after 8 h of incubation (Supplementary Fig. 7c). In contrast, within 8 h both Mistic-Alg3 and Mistic-Alg9 completed conversion reactions, indicating a slower reaction rate of Alg12 compared to the other two MTases (Supplementary Fig. 7). In yeast, Alg12 is N-glycosylated on one or more asparagines. Therefore, the weaker kinetic property of Alg12 may be attributed to its lack of N-glycan resulting from expression in *E. coli*.

Alg9 is predicted to add both the seventh and ninth α1,2-linked mannoses of M9GN2-PP-Phy^28^. In vitro extension of M9GN2 was performed by sequentially incubating M5GN2-PP-Phy with membrane fractions from *E. coli* expressing Mistic-Alg3, Mistic-Alg9, and Alg12. M5GN2-PP-Phy was incubated with membrane fractions from *E. coli* expressing Mistic-Alg3 for 12 h, after which Mistic-Alg9 added for an additional 12 h, then sequentially incubated with membrane fractions containing Alg12 and Mistic-Alg9 for 12 h, respectively. Peaks eluting at ~17.2 min showed an *m*/*z* value of 1906.55, assignable as [M9GN2+Na]^+^ in accord with the calculated molecular weight of M9GN2 (1883.67) (Fig. 3b, c). These data demonstrated that these sequential reactions produced M9GN2 from M5GN2 with a high conversion rate (88.6%) (Fig. 3b). Analysis of mannose linkages of M9GN2 was performed with linkage-specific mannosidases. As shown in Fig. 4d, treatment of M9GN2 with α1,2-mannosidase yielded M5A2BCGN2, as predicted for removal of two α1,2 mannoses from the A-arm, one α1,2-linked mannose from the B-arm, and one α1,2-linked mannose from the C-arm. Similarly, α1,2-3-mannosidase digestion removed four α1,2-linked mannoses and two additional α1,3-linked mannoses (from A-arm and B-arm, respectively) to generate M3A3B2CGN2 with two α1,6 mannoside linkages on C arm. Peaks corresponding to M1GN2 were observed after treatment with α1,2-3 and α1,6 mannosidases, the latter removing two additional α1,6 mannoses. Further treatment with β-mannosidase hydrolyzed the last β1,4-linked mannose, leaving GN2 as the sole product (Fig. 4d). Taken together, these results demonstrated unequivocally that M9GN2 bearing four α1,2-man, two α1,3-man, two α1,6-man, and one β1,4-man was successfully reconstituted in vitro, and its structure is identical to that produced in vivo^52^.

To efficiently synthesize the M9GN2 glycan from M5GN2-PP-Phy, a one-pot reaction was attempted. Membrane fractions prepared from *E. coli* expressing Mistic-Alg3, Mistic-Alg9, and Alg12 were mixed together and incubated with M5GN2-PP-Phy for 20 h. After acid hydrolysis, reaction products were subjected to UPLC–MS analysis. As shown in Fig. 3d, UPLC peaks corresponding to M5GN2 substrate (eluting at around 14.8 min) were completely converted to product in a single peak eluting at ~17.3 min. This one-pot reaction converted 100% of M5GN2 to M9GN2, an efficiency higher than that of its stepwise synthesis (Fig. 3c, d). The reason for this difference in yield was not further examined. Nevertheless, these results demonstrate the successful in vitro synthesis of M9GN2 with near complete yield, with a product yield of ~12 μg from 100 μL reaction volume. When scaled-up, this reaction gave milligram quantities of the product, whose structure was verified by mass analysis and NMR spectrum (see Supplementary Notes 1–3, Supplementary Figs. 4b, 5b–d).

### Alg MTases display strict substrate specificity

There is strong genetic evidence to support the model that the strict substrate specificity of each Alg GTase leads to ordered assembly of LLO glycans^23,29^. Our in vitro system provided an opportunity to rigorously test the specificity of each Alg MTases by varying the order of addition of each enzyme in the presence of different substrates.

**Fig. 5:**
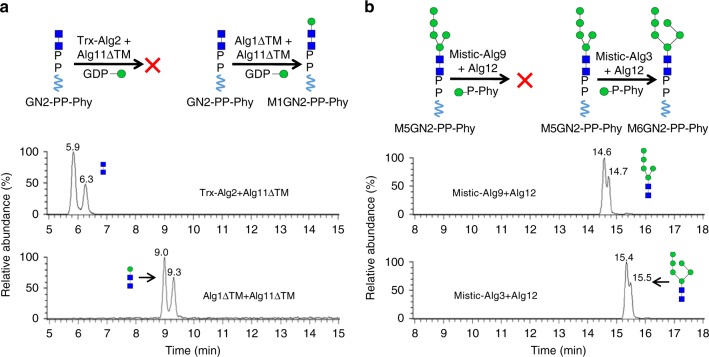
Substrate specificity of Alg MTases. **a** Substrate specificity of Alg1, Alg2, and Alg11. Reactions contained GN2-PP-Phy and GDP-Man and the indicated combination of Alg1∆TM, Trx-Alg2, and/or Alg11∆TM. Glycan products were analyzed by UPLC–MS. **b** Substrate specificity of Alg3, Alg9, and Alg12. Each reaction contained M5GN2-PP-Phy and Man-P-Phy and the indicated combination of Mistic-Alg3, Mistic-Alg9, and Alg12. Glycan products were analyzed by UPLC–MS. Reaction products are indicated by the arrows, and their deduced structure is depicted schematically

The cytosolic Alg MTases catalyze all the cytosolic-facing ER reactions. Among them, Alg1 is known to be responsible for the addition of a β1,4-linked mannose to GN2-PP-Phy. To determine if Alg2 or Alg11 could also use GN2-PP-Phy as substrate, Trx-Alg2 and Alg11ΔTM were incubated in the reaction buffer with GN2-PP-Phy and GDP-Man. Reaction products were analyzed after hydrolysis by UPLC–MS. As shown in Fig. 5a, in the absence of Alg1, no mannosylation product was detected, indicating that neither Trx-Alg2 nor Alg11ΔTM could recognize GN2 as the substrate. When Alg1ΔTM and Alg11ΔTM were incubated with GN2-PP-Phy and GDP-Man in the absence of Trx-Alg2, Alg1ΔTM extended GN2 to M1GN2, but M1GN2 was not further mannosylated by Alg11ΔTM (Fig. 5a). This result demonstrated that Alg11 could not recognize M1GN2-PP-Phy as its substrate. Taken together, these data demonstrate recombinant Alg1ΔTM, Trx-Alg2, and Alg11ΔTM each showed the capacity to distinguish the corresponding acceptor structures from other intermediates^23^.

Similar order of addition experiments was performed with Alg3, Alg9, and Alg12, which catalyze the luminal reactions to produce M9GN2 from M5GN2. Extension of M5GN2 was performed in the presence of M5GN2-PP-Phy and Man-P-Phy, along with different combinations of Alg3, Alg9, and Alg12. Reaction products were analyzed after hydrolysis by UPLC–MS (Fig. 5b). We found that in the absence of Alg3, neither Alg9 nor Alg12 could add any mannoses to M5GN2-PP-Phy. In the absence of Alg9, even after Alg3 extended M5GN2 to M6GN2, Alg12 could not add any additional mannoses to it (Fig. 5b). These results implied that Alg9 and Alg12 each are capable of recognizing only the product of preceding Alg in the LLO pathway. Consistent with in vivo specificity studies^29,53^, our results demonstrated that each of the recombinant Alg proteins display strict substrate specificity, and it is this substrate specificity that dictates the strict order of LLO mannosylation.

### Synthesis of non-physiological LLOs

**Fig. 6:**
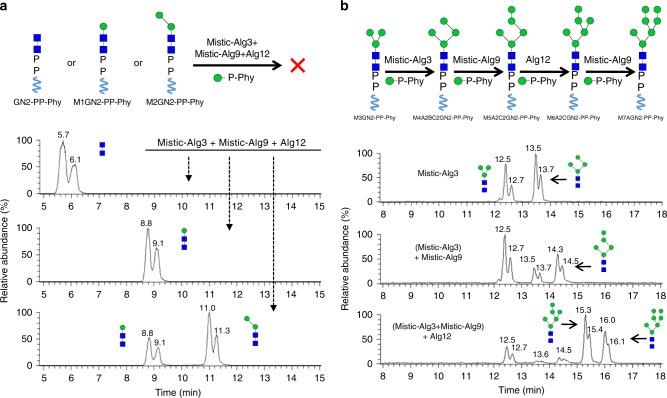
Specificity of the ER luminal MTases for non-physiological substrates. **a** Reactions were performed in the presence of GN2-PP-Phy, M1GN2-PP-Phy, or a mixture of M1GN2-PP-Phy/M2GN2-PP-Phy, and Man-P-Phy. Products were analyzed by UPLC–MS. Addition of Mistic-Alg3, Mistic-Alg9, and Alg12 (Mistic-Alg3+Mistic-Alg9+Alg12) failed to elongate any of those substrates. **b** Reactions were performed in the presence of M3GN2-PP-Phy and Man-P-Phy. Stepwise addition of the membrane fractions of Mistic-Alg3, Mistic-Alg9, and Alg12 produced a variety of unusual LLOs, whose deduced structure is shown schematically. Addition of Mistic-Alg3 alone generated M4A2BC2GN2 (Mistic-Alg3); sequential addition of Mistic-Alg3 and Mistic-Alg9 generated M5A2C2GN2 [(Mistic-Alg3)+Mistic-Alg9]; sequential addition of Mistic-Alg3, Mistic-Alg9, and Alg12 generated M6A2CGN2 and M7AGN2 [(Mistic-Alg3+Mistic-Alg9)+Alg12]. Products are indicated by the arrows

Another notable observation was the absence of unusual non-physiological oligosaccharide products in these in vitro reactions. In vivo, LLO synthesis is compartmentalized in the cytosol and lumen. These two topologically distinct reaction stages are bridged by a flippase that translocates M5GN2-PP-Dol across the membrane from the cytosol to the ER lumen (Fig. 1). Because of these topological constraints, MTases whose catalytic domains reside in the ER lumen or in the cytosol are exposed to only a subset of LLO intermediates in vivo. That is, cytosolic LLO intermediates are not found in the ER lumen and vice versa. Our in vitro two-pot reactions mimicked these constraints by physically separating the Alg1, Alg2, and Alg11 reactions from those of Alg3, Alg9, and Alg12. Thus, one explanation for the absence of unusual glycan byproducts is that their synthesis is simply prevented by compartmentalization of enzymes. If this is correct, luminal enzymes should be able to extend cytosolic intermediates if they are available. To test this idea, cytosolic LLOs, including GN2-PP-Phy, M1GN2-PP-Phy, M3GN2-PP-Phy, and an intermediate M2GN2-PP-Phy (Man-(α1,3)-Man-GlcNAc2-PP-Phy) generated by an Alg2 mutant (G257P) that accumulates M1GN2 and M2GN2 were prepared^25^. These intermediates were incubated with recombinant Alg3, Alg9, and Alg12, and glycan products analyzed by UPLC–MS. In the presence of Mistic-Alg3, Mistic-Alg9, and Alg12, neither GN2-PP-Phy, M1GN2-PP-Phy, nor M2GN2-PP-Phy was elongated (Fig. 6a). In contrast, a significant amount of M3GN2-PP-Phy was elongated by Alg3 to produce M4A2BC2GN2 (Fig. 6b). Two groups of peaks could be detected by UPLC–MS. As confirmed by MS, glycans in the first group eluted ~12.7 min and were derived from M3GN2-PP-Phy while glycans in the second group of peaks eluted at about 13.7 min and were derived from M4A2BC2GN2 (Supplementary Fig. 8a). Sequential addition of Mistic-Alg3 and Mistic-Alg9 converted M3GN2-PP-Phy to M5A2C2GN2-PP-Phy. In contrast, incubation of M3GN2 with both Mistic-Alg9 and Alg12 failed to produce any additional products. These results suggested that extension of M3GN2-PP-Phy to M5A2C2GN2-PP-Phy requires successive and ordered mannosylation by Alg3 and Alg9. This idea was verified since a reaction that included M3GN2 incubated sequentially with Mistic-Alg3 for 12 h, followed Mistic-Alg9 for another 12 h, followed by Alg12 for another 12 h, produced M7AGN2 (Fig. 6b). The acid-hydrolyzed glycan products were analyzed by UPLC–MS (Supplementary Fig. 8a). To confirm the structures of these unusual LLOs, the final product M7AGN2 was treated with specific mannosidases (Supplementary Fig. 8b). Our results confirmed that all the additional mannoses added on M3GN2 in M7AGN2 derived from the ordered mannosylation by Alg3, Alg9, and Alg12. Therefore, we concluded that M3GN2 can serve as the substrate of ER luminal MTases in vitro for production of several unusual LLOs bearing high-mannose type glycans (Supplementary Table 1).

## Discussion

In this study, we describe in vitro reconstitution of the eukaryotic LLO pathway from GN2 to M9GN2, using recombinant Alg MTases. Reconstitution involved two successive one-pot reactions, which correspond to the in vivo cytosolic and luminal reactions. First, five mannoses were added to GN2 by recombinant Alg1, Alg2, and Alg11 to produce M5GN2. This M5GN2 then served as the substrate for extension by recombinant Alg3, Alg9, and Alg12 to produce M9GN2. These two sequential one-pot reactions produced an M9GN2 oligosaccharide whose mannose linkages were verified to be identical to that produced in vivo.

In vitro synthesis of M5GN2 was first described by Locher and co-workers using yeast Alg1, Alg11, and human Alg2 expressed and purified from mammalian cells^43^. While this method provides proof of principle, it is impractical as general strategy for M5GN2 production because of its low yield, as well as the high cost of preparing human Alg2. We previously described purification of active, recombinant yeast Alg2 from *E. coli* and built on that approach for M5GN2 synthesis in the present study. Mannosylation of GN2-PP-Phy by purified Alg1ΔTM, Trx-Alg2, and Alg11ΔTM to produce M5GN2-PP-Phy was extremely efficient, with a conversion rate approaching 100% (Fig. 2b). Alg1, Alg2, and Alg11 associate in vivo^46^ and we discovered they also do so in *E. coli* when co-expressed. This property was exploited to develop a one-pot reaction to mannosylate GN2-PP-Phy to M5GN2-PP-Phy, with a membrane fraction from *E. coli* that simultaneously over-expressed recombinant Alg1, Alg2, and Alg11 as a source of enzymes (Fig. 2c). This co-expression system is important because it significantly simplifies M5GN2-PP-Phy synthesis in vitro.

Assembly of M9GN2-PP-Dol from M5GN2 occurs in the ER lumen, and genetic evidence has implicated Alg3, Alg9, and Alg12 as the GTases responsible for these activities^3^. Our experiments showing that recombinant Mistic-Alg3, Mistic-Alg9, and Alg12 were both necessary and sufficient to extend M5GN2 to M9GN2 provide direct evidence for their activities, which has thus far been lacking (Figs. 3, 4). Furthermore, the substrate specificity of each of these MTases was studied by adding different combinations of Alg proteins to the substrate (Fig. 5). Our results not only provided direct evidence that Alg3, Alg9, and Alg12 are the luminal MTases that elongate M5GN2 to M9GN2 during LLO biosynthesis in vivo, but also verified the strict substrate specificity of their MTase activities at enzyme level (Fig. 5b).

Overexpression of Alg12 in an *alg9Δ* strain accumulates similar levels of M6GN2 and M7BCGN2 N-glycans in vivo, indicating that the second α1,6-man (Fig. 1) can be directly added to the M6GN2 structure even in the absence of Alg9 in vivo^54,55^. We did not observe such a direct modification on M6GN2 by recombinant Alg12 in vitro under standard conditions (Fig. 5b). However, addition of a five-fold excess of Alg12 coupled with a much longer incubation time (to over 48 h) allowed detection of a small amount of product (Supplementary Fig. 9a). This product was isolated and analyzed by UPLC–MS, and treated with a series of linkage-specific mannosidases as described above (Supplementary Fig. 9b, c). These experiments confirmed this product as M7BCGN2, which has an additional α1,6-man on the C arm of M6GN2. One explanation for the apparent difference between the in vivo and in vitro reactions may be due to a more robust activity of N-glycosylated Alg12 produced in a eukaryote compared to recombinant Alg12 from *E. coli* (Supplementary Fig. 7c) that lacks N-glycan(s).

Interestingly, stepwise synthesis of M9GN2 using Mistic-Alg3, Mistic-Alg9, and Alg12 from membrane fractions converted <90% of M5GN2-PP-Phy to M9GN2-PP-Phy (Fig. 3b), while the one-pot reaction with membranes showed ~100% conversion. This suggests that these luminal MTases work better when simultaneously mixed. The cytosolic-facing Alg1, Alg2, and Alg11 MTases (Fig. 2c) work as a multimeric complex in vivo^46^ and it is tempting to speculate that the luminal Alg3, Alg9, and Alg12 may physically interact with one another as well. This physical interaction would be facilitated in the one pot reaction, which in turn may promote the higher efficiency we observe in vitro. Further experiments are required to test if Alg3, Alg9, and Alg12 form complexes in vivo or in vitro.

In vivo, M3GN2-PP-Dol cannot gain access to the ER lumen. This creates a topological constraint that prevents the luminal Alg3, Alg9, and Alg12 from potentially producing aberrant LLO products. When M3GN2-PP-Phy was incubated with recombinant Alg3, we observed a moderate yield of M4A2BC2GN2 glycan (Fig. 6b), suggesting that the two Alg11-catalyzed α1,2-mannose on the A arm are not critical ligands required for glycan recognition by Alg3. Further mannosylation of M4A2BC2GN2 by Alg9 produced M5A2C2 glycan, and the combination of Alg3, Alg9, and Alg12 resulted in M7AGN2 (Fig. 6b). Interestingly, incubation with the combination of Alg3, Alg9, and Alg12 somehow increased the conversion rate of Alg3, leading to the accumulation of M6A2CGN2 and M7AGN2. *alg11*Δ yeast mutants have been reported to accumulate some unusual LLO with six or seven mannoses^26,56^, but how or why this happens has been a mystery. Our results provide an explanation of this phenomenon. *alg11∆* mutants accumulate high levels of M3GN2-PP-Dol. This may favor the occasional translocation of M3GN2 into the ER lumen, which in turn could provide the M3GN2-PP-Dol substrate for elongation by Alg3, Alg9, and Alg12 to produce aberrant luminal LLOs. The ability to produce these unusual high-mannose glycans, such as M7AGN2 (Supplementary Table 1), may be useful for biochemical studies that will provide a deeper understanding of the substrate specificity of these MTases.

Scaling-up these two one-pot reactions produced milligram quantities of products, which are sufficient for performing further bioassays. M9GN2 is a crucial glycan intermediate in the N-linked glycosylation pathway as the substrate for downstream enzymes involved in its transfer to protein and further modification. Thus, our study provides an important tool for biochemical studies of the mechanism and structure of those downstream enzymes.

## Methods

### Plasmid constructions

All expression plasmids were constructed such that each encoded Alg protein contained an N-terminal His6 tag. pET28-Alg1ΔTM and pET32-Trx-Alg2 have been described^25,45^. The Mistic gene^49^, was synthesized (BGI, Shenzhen, China) and cloned into the *Nde*I and *Nhe*I sites of pET28 (Thermo Scientific, MA, USA), generating pET28-Mistic. Yeast genes encoding Alg11ΔTM (aa 45-548), Dpm1, Alg3, Alg9, and Alg12 were amplified by PCR using genomic DNA of *S. cerevisiae*. *ALG11ΔTM*, *DPM1*, and *ALG12* were cloned into vector pET28. *ALG3* and *ALG9* were cloned into pET28-Mistic. Oligonucleotide primers are listed in Supplemetary Table 2. Expression plasmids, including their description, parent plasmid, and cloning sites are listed in Supplementary Table 3. Plasmid sequencing data are provided as Supplementary Data 1. To construct the Alg1, Alg2, Alg11 co-expression plasmid (pET28-Alg1∆TM-Trx-Alg2-Alg11∆TM), *ALG1ΔTM*, *TRX*-*ALG2*, and *ALG11ΔTM* were amplified by PCR from pET28-Alg1∆TM, pET32-Trx-Alg2, and pET28-Alg11ΔTM, and sequentially cloned into *Nco*I and *BamH*I, *Sac*I and *EcoR*I, *Not*I and *Xho*I sites of one pET28 vector, respectively. Insertion of 5′ T7 promoter and ribosome-binding sites in front of both *TRX*-*ALG2* and *ALG11ΔTM* genes allowed their simultaneous expression.

### Protein expression and purification

All proteins used in this study contained an N-terminal His6-tag. Overexpression of Alg1ΔTM, Trx-Alg2, Alg11ΔTM, Dpm1, Mistic-Alg3, Mistic-Alg9, Alg12, and the co-expression of Alg1∆TM, Trx-Alg2, and Alg11∆TM were performed in *E. coli* Rosetta (DE3) cells originating from BL21 (Thermo Scientific, MA, USA, Catalog Number: 70-954-4). Cells were cultured in Terrific-Broth (TB, 1.2% tryptone, 2.4% yeast extract, 0.5% glycerol, 17 mM KH_2_PO_4_, and 72 mM K_2_HPO_4_) at 37 °C until the OD_600_ was between 0.8 and 1.2. Cultures were cooled to 16 °C prior to induction with isopropyl-β-d-thiogalactopyranoside (IPTG, Sangon Biotech, Shanghai, China). IPTG was added to a final concentration of 0.1 mM to induce T7-dependent gene expression. Cultures were induced overnight with shaking at 16 °C. After induction, cells were harvested and resuspended in buffer A [25 mM Tris/HCl (pH 8.0) and 150 mM NaCl], then disrupted by sonication on ice to produce a lysate that was further processed as described below.

Dpm1 was purified using the same method as previously described for purification of Alg1ΔTM and Trx-Alg2^25,45^. Briefly, the cell lysate was spun down to remove cellular debris (4000×*g*, 20 min, 4 °C), followed by pelleting of insoluble materials containing *E. coli* membrane (20,000×*g*, 90 min, 4 °C). Proteins in the insoluble fraction were solubilized for 1 h in buffer A containing 1% Triton X-100. His-tagged Dpm1 was purified from the detergent-soluble fraction with HisTrap HP affinity chromatography (GE Healthcare, Buckinghamshire, UK). Alg11ΔTM was purified as followed. The cell lysate was spun down to remove cellular debris and insoluble material (12,000×*g*, 30 min, 4 °C). Alg11ΔTM in the supernatant was purified using HisTrap HP affinity chromatography. The purified protein was dialyzed against buffer [25 mM Tris–HCl (pH 8.0), 50 mM NaCl], followed by concentration using Amicon Ultra 10K NMWL filtration units (Millipore, MA, USA). Protein concentration was determined with the BCA assay kit (Sangon Biotech, Shanghai, China).

To prepare membrane fractions from *E. coli* expressing recombinant proteins, the cell lysate was spun down to remove cellular debris (4000×*g*, 20 min, 4 °C), followed by pelleting of the membranes (100,000×*g*, 60 min, 4 °C). For Mistic-Alg3, Mistic-Alg9, and Alg12, membranes were homogenized in [14 mM MES/NaOH (pH 6.5), 30% glycerol]; for co-expressed Alg1∆TM, Trx-Alg2, and Alg11∆TM, membranes were homogenized in [50 mM Tris/HCl (pH 7.5), 30% glycerol]. The membrane fractions were stored at −20 °C. Enzyme activity in membranes remained active for at least 3 months.

Protein expression was verified by sodium dodecyl sulfate–polyacrylamide gel electrophoresis (SDS–PAGE) and western blotting (Supplementary Fig. 1). Protein samples were boiled for 5 min at 100 °C before loading. Typically, 10 µg of protein samples were separated by 10% or 12% SDS–PAGE, followed by coomassie brilliant blue staining. For western blotting, samples were transferred onto polyvinylidenedifluoride membranes (Bio-Rad, CA, USA), incubated with anti-His mouse mAb (1:2000) (Code number: HT501, Lot number: M21022, TransGen Biotech, Beijing, China), followed by goat anti-mouse IgG, HRP (1:5000) (Code number: H5201-01, Lot number: M21015, TransGen Biotech, Beijing, China) and detected by chemiluminescence (ECL, Bio-Rad, CA, USA).

### Preparation of phytanyl phosphate mannose with Dpm1

Chemo-enzymatic synthesis of phytanyl phosphate mannose (Man-P-Phy) was performed as reported^51^. Briefly, standard reaction mixtures contained 50 mM Tris/HCl (pH 7.5), 10 mM MgCl_2_, 1% NP-40, 20 mM P-Phy, 50 mM GDP-Man (Sigma-Aldrich, MO, USA), and 2 mg/mL purified Dpm1 in a total volume of 50 µL. The reaction was performed at 30 °C for 10 h, Dpm1-dependent mannose transfer efficiency from GDP-Man to P-Phy was monitored by thin layer chromatography using Merck 60 F_254_ silica-coated plates (Millipore, MA, USA) with a chloroform/methanol/water (6.5:3.5:0.4, V/V) solvent and developed in ethanol/sulfuric acid (19:1, V/V) with heating. The conversion yield of P-Phy to Man-P-Phy was estimated by comparing the newly formed product spot with P-Phy. This reaction mixture was directly added into MTase reactions without further treatment.

### Enzymatic assembly of M1~9GN2-PP-Phy

The chemical synthesis of GN2-PP-Phy was prepared as reported^57,58^. All enzyme assays were performed in the following buffer: [14 mM MES/NaOH (pH 6.0), 4 mM potassium citrate, 10 mM MgCl_2_, 10 mM MnCl_2_, 0.05% NP-40, 50 μM GN2-PP-Phy, 1 M sucrose; 2 mM GDP-Man] in a total volume of 100 µL. For assembly of the M5GN2-PP-Phy, purified enzymes [Alg1ΔTM (0.5 µg/mL), Trx-Alg2 (150 µg/mL), and Alg11ΔTM (50 µg/mL)] were added stepwise and incubated at 30 °C for 12 h (each); Reactions performed with membrane fractions from *E. coli* that co-expressed Alg1ΔTM, Trx-Alg2, and Alg11ΔTM (20 µg/mL) were incubated at 30 °C for 12 h.

For stepwise assembly of M9GN2-PP-Phy from M5GN2-PP-Phy, membrane fractions from *E. coli* expressing [Mistic-Alg3 (20 mg/mL), Mistic-Alg9 (20 mg/mL), and Alg12 (20 mg/mL)] were added stepwise and incubated at 30 °C for 12 h with 2 mM Man-P-Phy. For one-pot synthesis of M9GN2-PP-Phy from M5GN2-PP-Phy, these membrane fractions were incubated simultaneously with 2 mM Man-P-Phy at 30 °C for 20 h, rather than sequentially.

### Mannosidase digestions

Digestion of glycans (2.5 nmol) with 3.2 U of α1,2-3-mannosidase (*Xanthomonas manihotis*, New England Biolabs, MA, USA), 4 U of α1,6-mannosidase (*X. manihotis*, New England Biolabs), and 0.1 mU of α1,2-mannosidase (*Aspergillus saitoi*, ProZyme, CA, USA) were performed in 10 μL at 25 °C for 16 h in buffers supplied by the manufacture. Digestion of glycans (2.5 nmol) with 25 mU of β-mannosidase (*Helix pomatia*, Sigma-Aldrich, MO, USA) were performed in 50 mM sodium citrate (pH 4.4) at 25 °C for 16 h.

### UPLC–MS analysis of saccharides

Samples were hydrolyzed with 20 mM hydrogen chloride. After 1 h incubation at 100 °C, the water-soluble glycan-containing fraction was desalted by solid-phase extraction using 1 mL Supelclean ENVI-Carb Slurry (Sigma-Aldrich, MO, USA) and lyophilized. Dried samples were dissolved in water prior to UPLC–MS analysis. Samples were analyzed on a TSQ Quantum Ultra (Thermo Scientific, MA, USA) coupled to a Dionex Ultimate 3000 UPLC (Thermo Scientific, MA, USA). Glycans were applied on an Acquity UPLC BEH Amide column (1.7 μm, 2.1 × 100 mm, Waters, MA, USA) and eluted with an acetonitrile gradient with a flow rate 0.2 mL/min. The gradient program was set as follows; 0–2 min, isocratic 80% acetonitrile; 2–15 min, 80–50% acetonitrile; 15–18 min, isocratic 50% acetonitrile. Eluent was monitored by measuring total ions at positive mode in the mass range of *m*/*z* 400–2000.

### Reporting summary

Further information on research design is available in the Nature Research Reporting Summary linked to this article.

## Supplementary information

Supplementary Information

Peer Review File

Description of Additional Supplementary Files

Supplementary Data 1

Reporting Summary

Source Data

## Data Availability

The source data underlying Supplementary Figs. 1a–d, 6b, c and 7a–c are provided as a Source Data file. A reporting summary for this Article is available as a Supplementary Information file. All other data that support the results of this study are available from the corresponding authors upon reasonable request.
