# Supplementary material for: Reconstitution of the lipid-linked oligosaccharide pathway for assembly of high-mannose N-glycans
Source: Nat Commun. 2019 Apr 18;10:1813. doi: 10.1038/s41467-019-09752-3 (PMC6472349; doi:10.1038/s41467-019-09752-3)
Supplement: Supplementary file 1 — Supplementary Information [file 41467_2019_9752_MOESM1_ESM.pdf]

Supplementary Information

Reconstitution of the lipid-linked oligosaccharide pathway for assembly of high-mannose *N*-glycans

Li *et al.*

Table of Contents

Supplementary Figures 1 to 9

Supplementary Tables 1-3

Supplementary Methods

Supplementary Notes 1-3

Supplementary References

Supplementary Figures

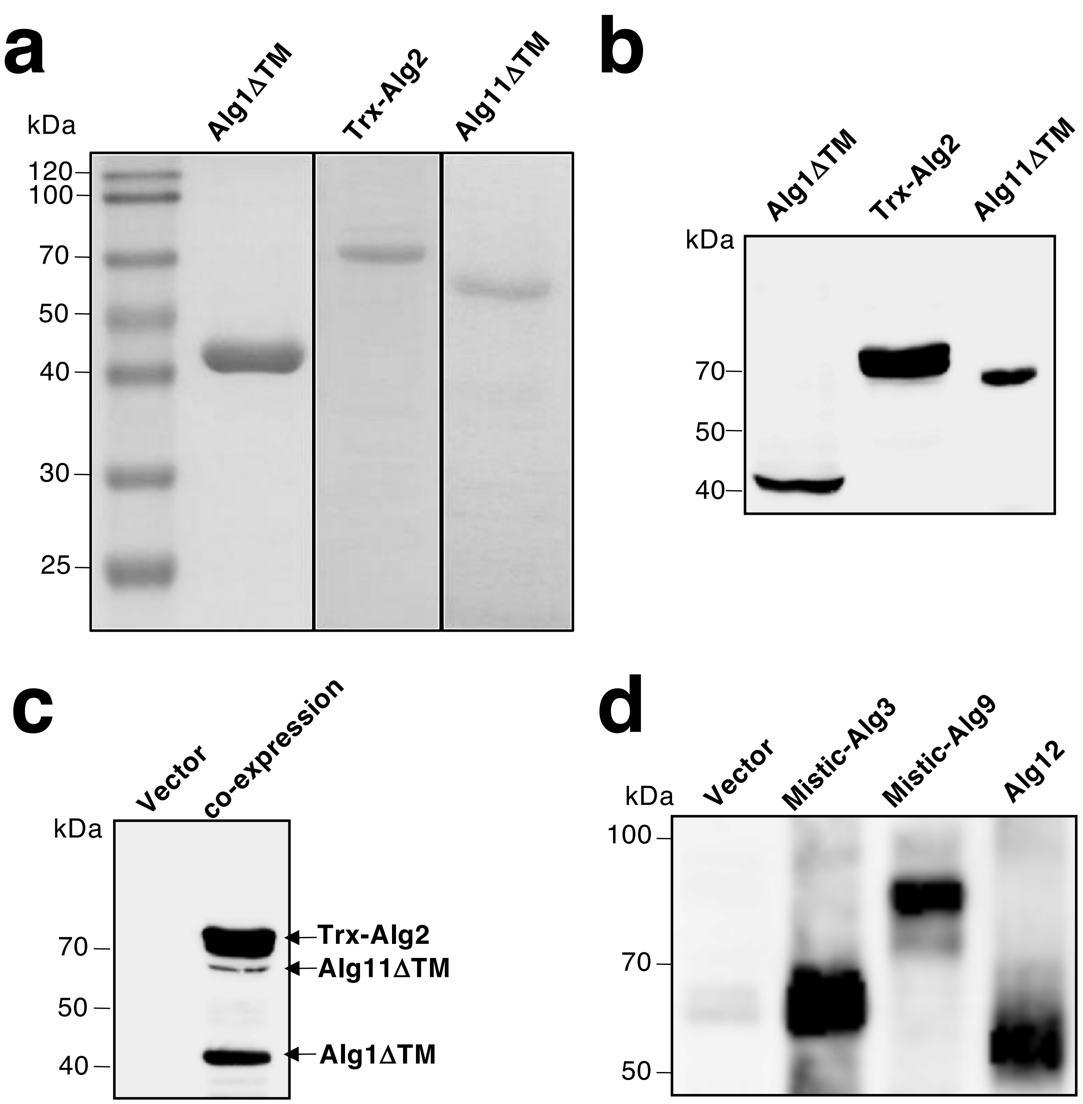

Supplementary Figure 1. (a) SDS-PAGE analysis of purified Alg1ΔTM, Trx-Alg2 and Alg11ΔTM. Purified proteins were separated by 12% SDS-PAGE gel, followed by staining with Coomassie Brilliant Blue R-250. (b) Western blot of purified His-tagged Alg1ΔTM, Trx-Alg2, Alg11ΔTM. Proteins were separated by 12% SDS-PAGE and immunoblotted with anti-His antibody. (c) Western blot of His-tagged Alg1ΔTM, Trx-Alg2 and Alg11ΔTM simultaneously co-expressed in *E. coli*. Ten μg of *E. coli* membrane fraction was separated by 10% SDS-PAGE and immunoblotted with anti-His antibody. (d) Western blot of His-tagged Mistic-Alg3, Mistic-Alg9 and Alg12. Equal amounts (10 μg) of *E. coli* membrane fractions were separated by 12% SDS-PAGE and immunoblotted with anti-His antibody. Source data are provided as a Source Data file.

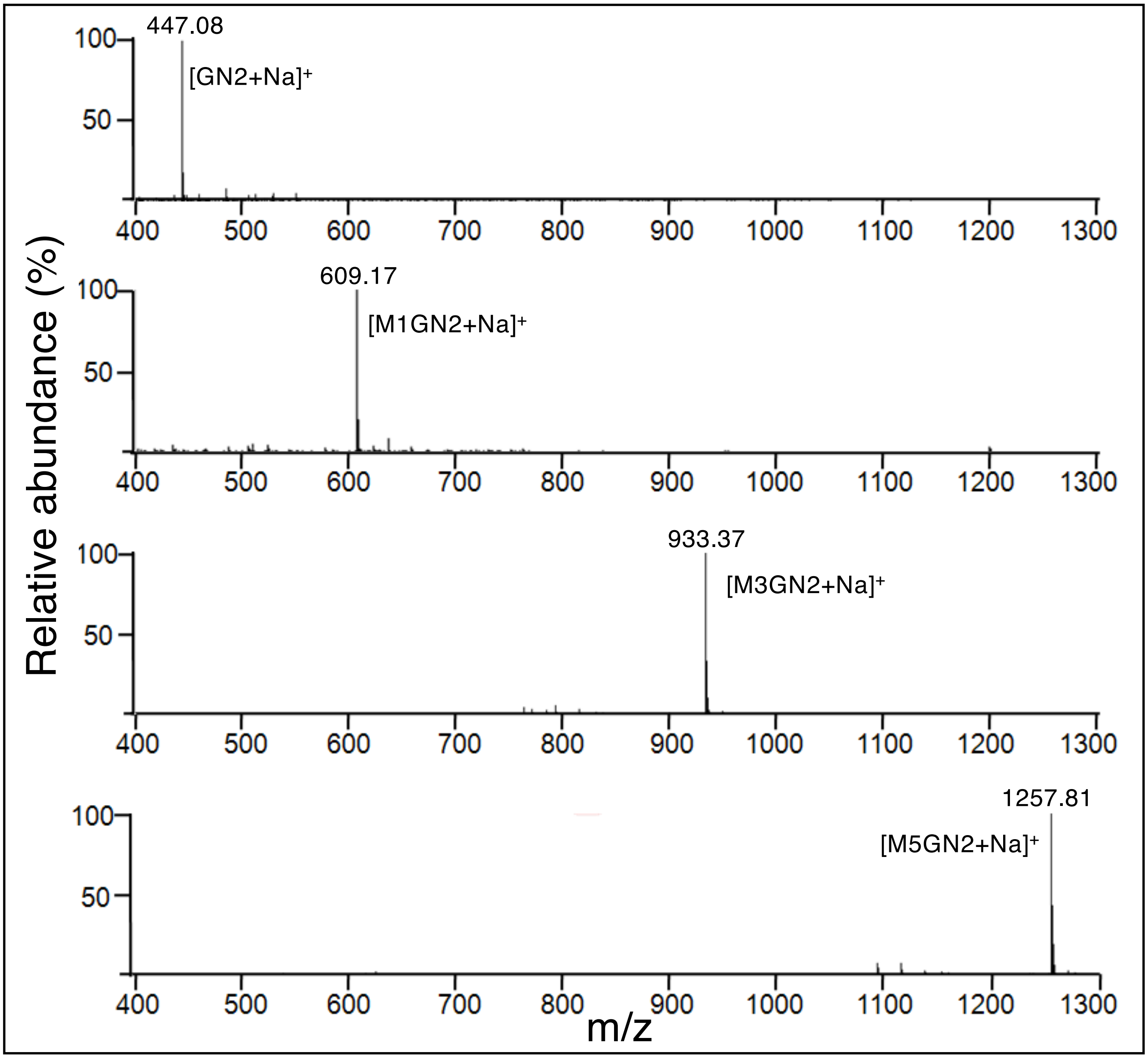

Supplementary Figure 2. Mass spectra of glycans released from LLOs. Mass analyses showed the peaks that eluted (Figure 2b) at ~6.1 min, ~9.1 min, ~12.7 min and ~14.8 min correspond to GN2 ($[GN2+Na]^+$), M1GN2 ($[M1GN2+Na]^+$), M3GN2 ($[M3GN2+Na]^+$) and M5GN2 ($[M5GN2+Na]^+$), respectively.

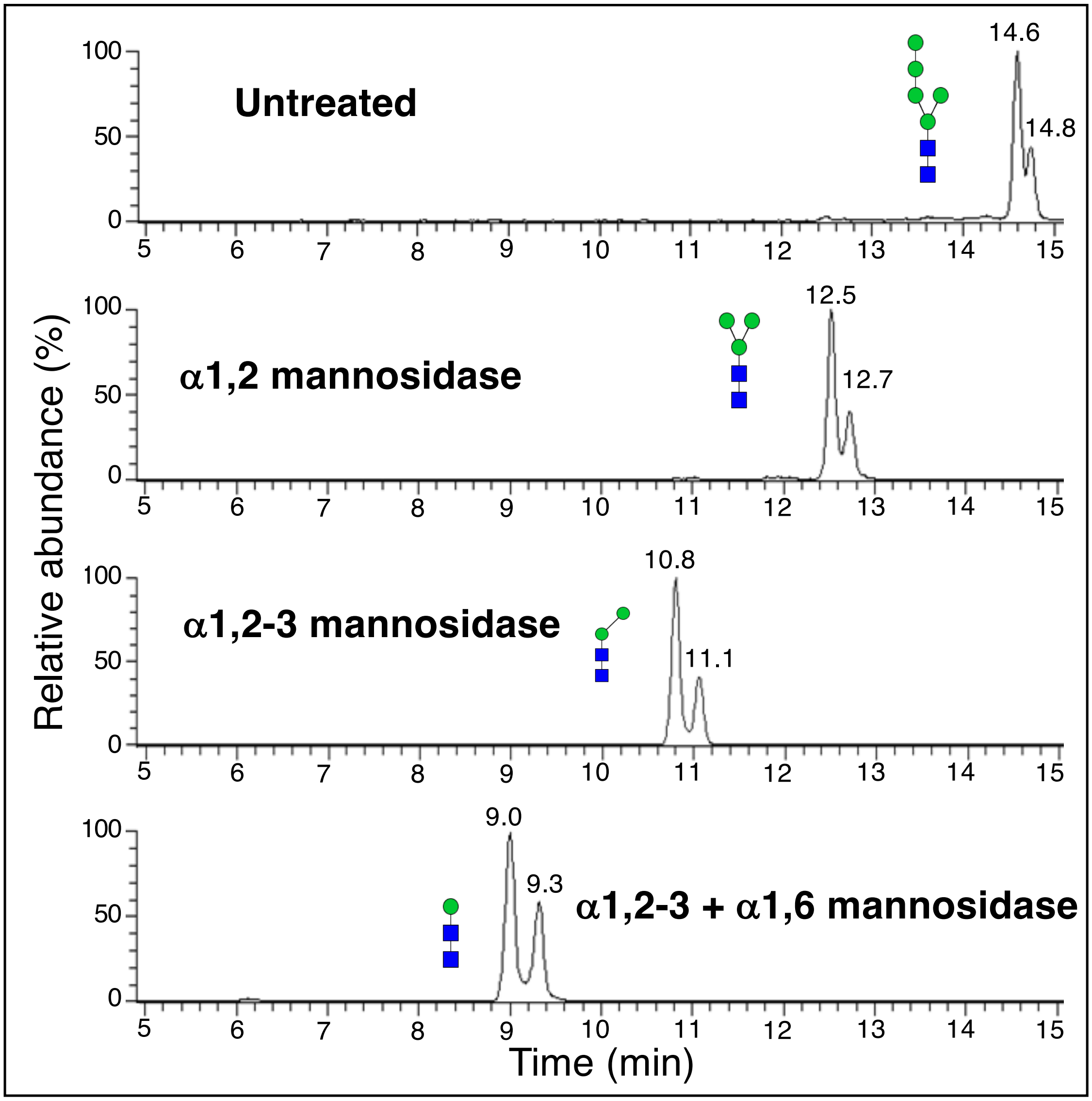

Supplementary Figure 3. **Mannosidase digestion of M5GN2 analyzed by UPLC-MS.** M5GN2 were digested with linkage-specific mannosidases, including: α1,2-mannosidase, which removes terminal α1,2 mannoses; α1,2-3-mannosidase, which removes terminal α1,2 mannoses and terminal α1,3 mannoses; α1,6-mannosidase, which removes terminal non-branched α1,6 mannoses. Digestion products and their deduced structure are depicted schematically: digestion of M5GN2 with α1,2-mannosidase produced M3GN2; with α1,2-3-mannosidase produced M2AGN2; while its digestion with α1,2-3-mannosidase and α1,6-mannosidase produced M1GN2.

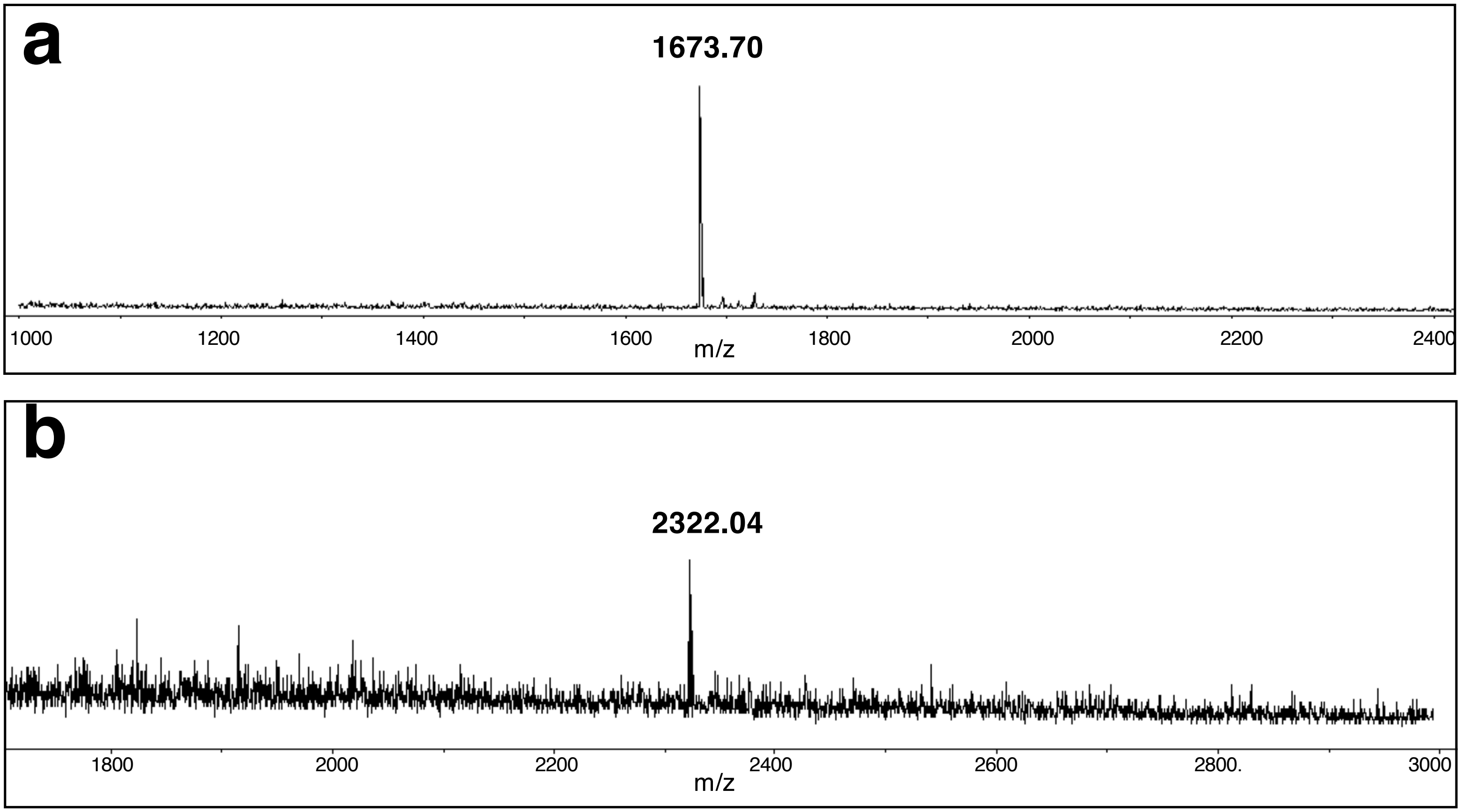

Supplementary Figure 4. *In situ* MALDI-TOF mass spectra of LLOs. The mass analyses were performed in the negative mode. (a) Mass spectrum of the reaction system to prepare M5GN2-PP-Phy. (b) Mass spectrum of the reaction system to prepare M9GN2-PP-Phy.

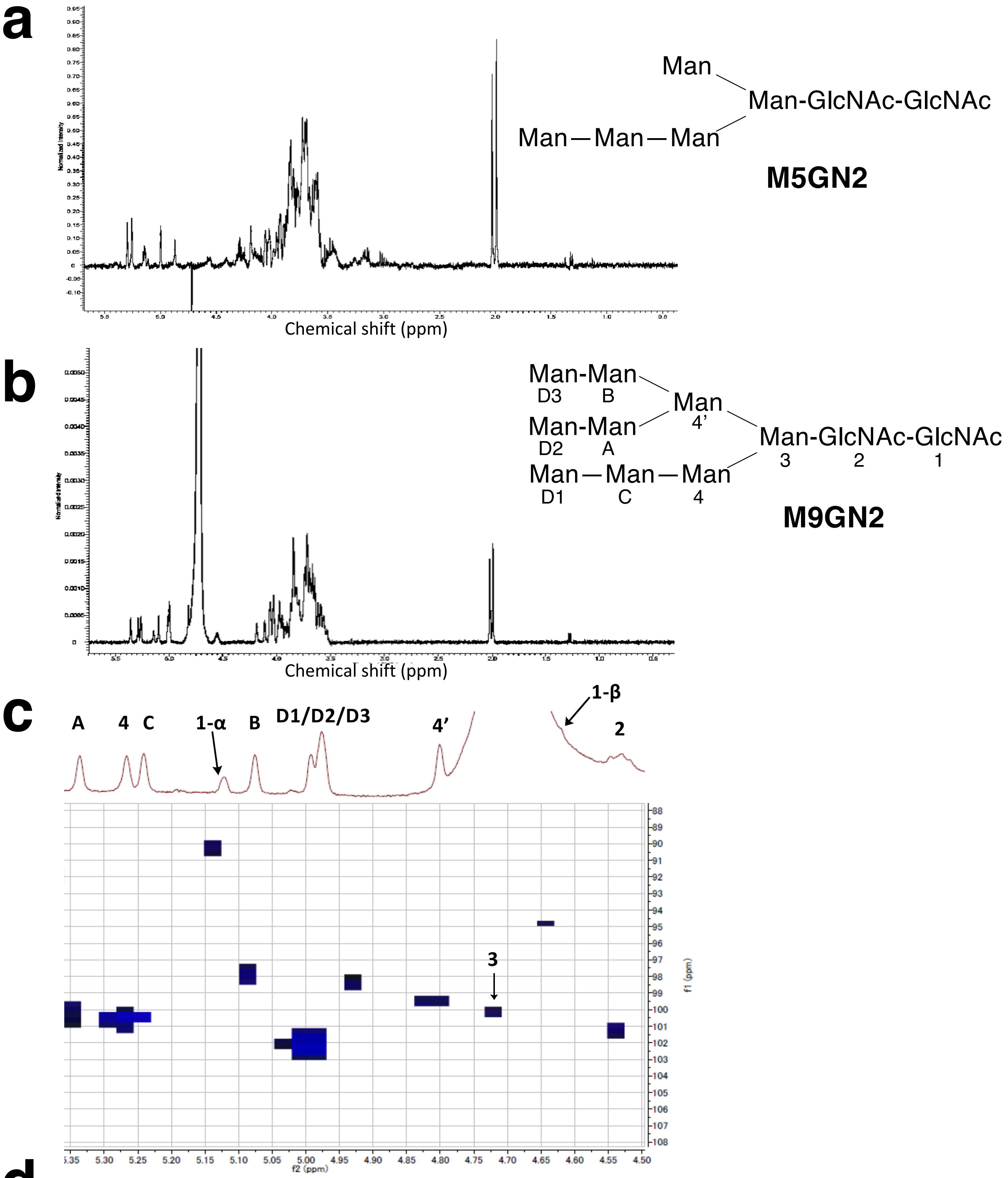

d

	GlcNAc		Man								
	1	2	3	4	4'	A	B	C	D1, D2, D3		
M9GN2 (α, this work)	5.14	4.55	4.73	5.29	4.82	5.36	5.10	5.26	5.00	5.00	5.01
M9GN2 (α)[1]	5.18	4.59	4.79	5.33	4.86	5.40	5.14	5.30	5.04	5.04	5.05
M9GN2-OC_3H_7 (β)[3]	4.36	4.45	---	5.20	4.73	5.27	5.01	5.17	4.90	4.90	4.92

Supplementary Figure 5. **NMR spectra of M5GN2 and M9GN2.** (a) Full range ^{1}H NMR spectrum of M5GN2. (b) Full range ^{1}H NMR spectrum of M9GN2. (c) Expanded spectrum of anomeric region in HSQC of M9GN2. (d) Chemical shift comparison of the anomeric protons in M9GN2 (this work) with reported data.

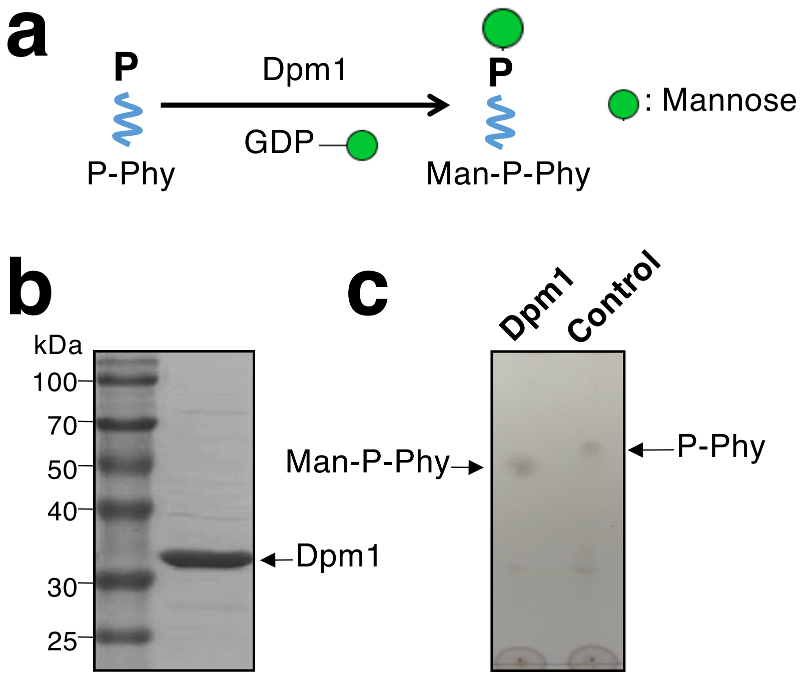

Supplementary Figure 6. **Dpm1-catalyzed Man-P-Phy synthesis.** (a) Schematic diagram of Man-P-Phy synthesized by Dpm1. (b) Purified Dpm1 was analyzed by 12% SDS-PAGE, followed by Coomassie Brilliant Blue R-250 staining. (c) TLC analysis of substrate P-Phy, indicated by the arrow, and products from a reaction containing P-Phy, GDP-Man and purified Dpm1. The Man-P-Phy product is indicated by the arrow. Source data are provided as a Source Data file.

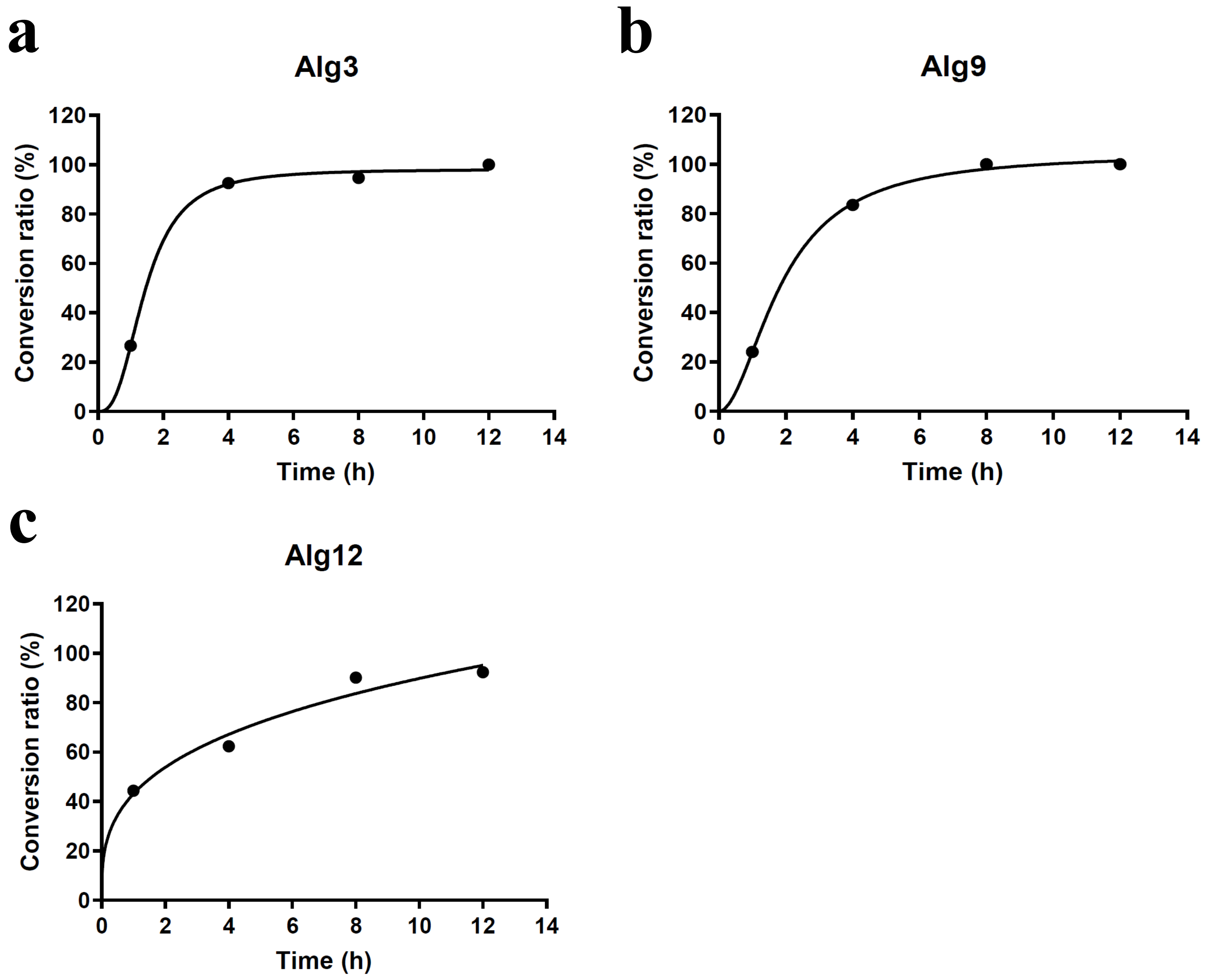

Supplementary Figure 7. **Kinetics of Mistic-Alg3, Mistic-Alg9 and Alg12.** Enzyme kinetics curves (GraphPad Prism 7.04) of these three MTases were drawn by measuring the conversion ratio of each reaction at different time points, i.e. 1 h, 4 h, 8 h and 12 h. (a) Kinetics of Mistic-Alg3. Membrane fractions from *E. coli* expressing Mistic-Alg3 (20 mg/mL) were added to M5GN2-PP-Phy (50 μM) and incubated at 30 ^{o}C with 2 mM Man-P-Phy; (b) Kinetics of Mistic-Alg9. Membrane fractions from *E. coli* expressing Mistic-Alg9 (20 mg/mL) were added to M6GN2-PP-Phy (50 μM) and incubated at 30 ^{o}C with 2 mM Man-P-Phy; (c) Kinetics of Alg12. Membrane fractions from *E. coli* expressing Alg12 (20 mg/mL) were added to M7GN2-PP-Phy (50 μM) and incubated at 30 ^{o}C with 2 mM Man-P-Phy. Source data are provided as a Source Data file.

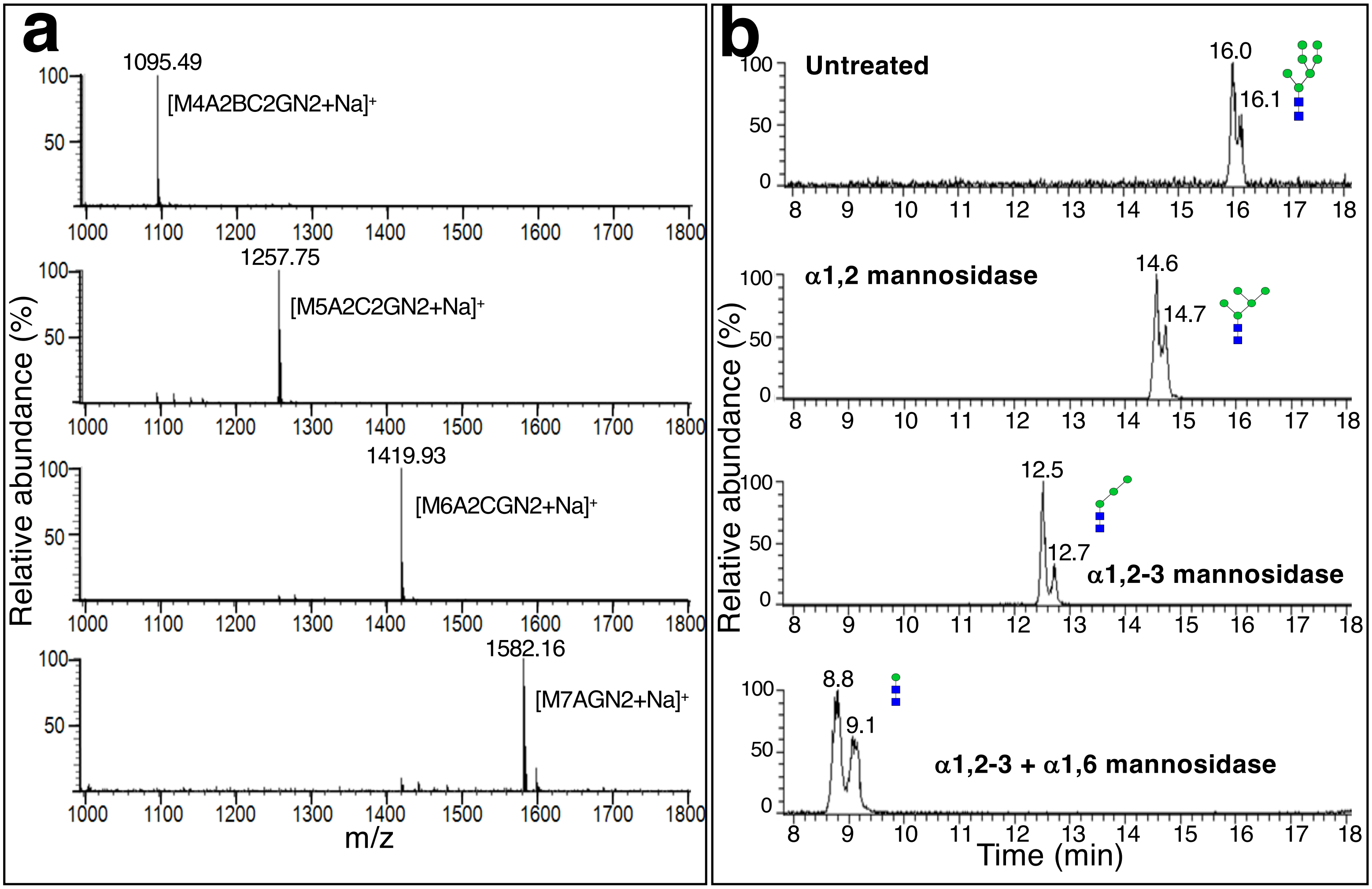

Supplementary Figure 8. (a) Mass spectra of glycans released from LLOs. Mass analyses showed the peaks eluted (Figure 6b) at ~13.7 min, ~14.5 min, ~15.4 min and ~16.1 min correspond to M4A2BC2GN2 ($[M4A2BC2GN2+Na]^+$), M5A2C2GN2 ($[M5A2C2GN2+Na]^+$), M6A2CGN2 ($[M6A2CGN2+Na]^+$) and M7AGN2 ($[M7AGN2+Na]^+$), respectively. (b) UPLC fraction (~16.1 min, Figure 6b) containing M7AGN2 was collected and treated with mannosidases, then analyzed by UPLC-MS. M7AGN2 were digested with linkage-specific mannosidases, including: α1,2-mannosidase, which removes terminal α1,2 mannoses; α1,2-3-mannosidase, which removes terminal α1,2 mannoses and terminal α1,3 mannoses; α1,6-mannosidase, which removes terminal non-branched α1,6 mannoses. Digestion products and their deduced structure are depicted schematically: digestion of M7AGN2 with α1,2-mannosidase produced M5A2BCGN2; with α1,2-3-mannosidase produced M3A3B2CGN2; while its digestion with α1,2-3-mannosidase and α1,6-mannosidase produced M1GN2.

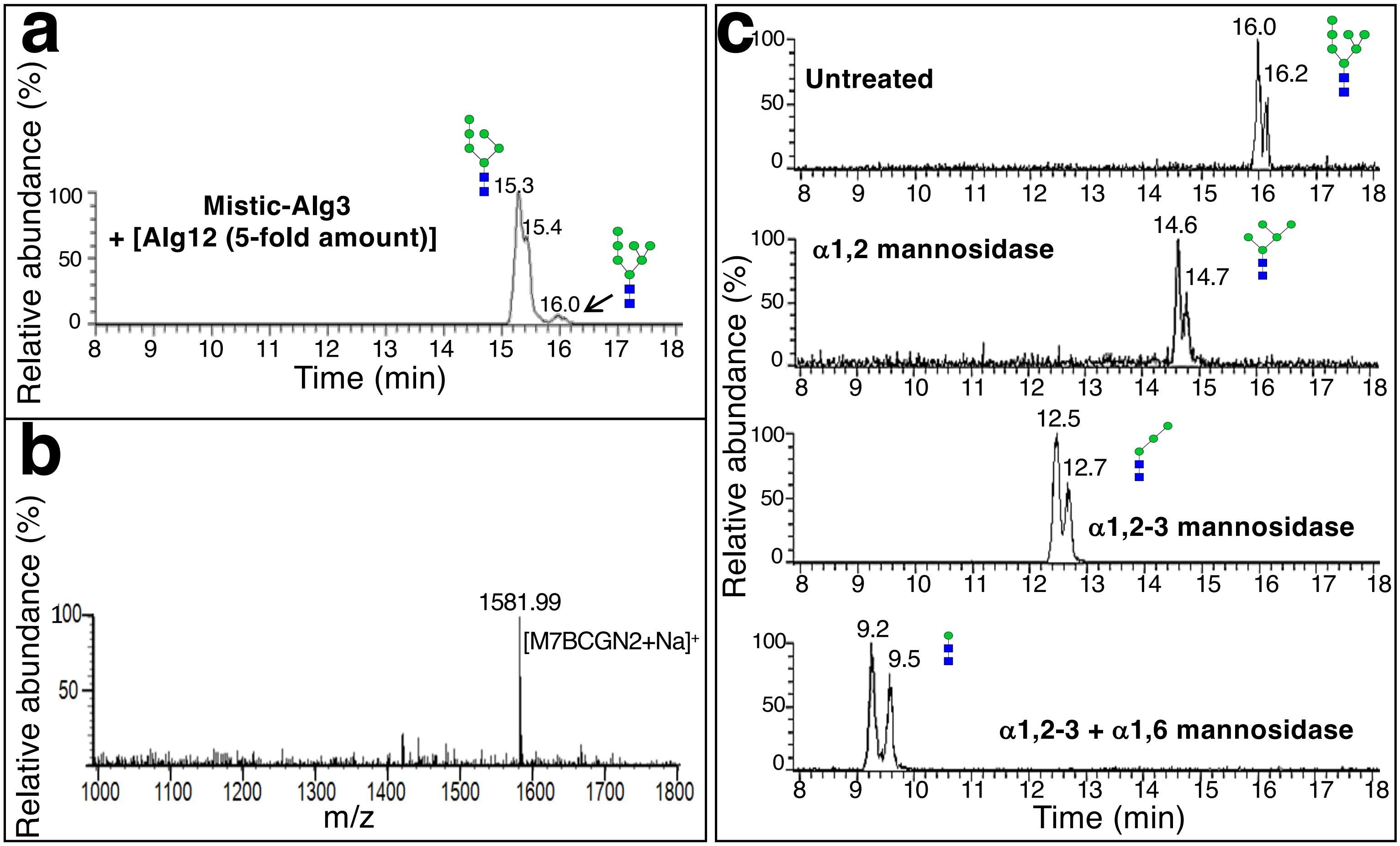

Supplementary Figure 9. **Trace amount of M7BCGN2-PP-Phy was produced with excess Alg12 *in vitro*.** (a) UPLC chromatogram of hydrolyzed glycans from the reaction with membrane fractions from *E. coli* expressing either Mistic-Alg3 or Alg12. In the presence of M5GN2-PP-Phy and Man-P-Phy, addition of Mistic-Alg3 produced M6GN2, sequential addition of 5-fold Alg12 and incubated for 48 h resulted in trace amount of M7BCGN2-PP-Phy. (b) Mass spectra of the glycan released from M7BCGN2-PP-Phy. Mass analyses showed the peaks eluted at ~16.0 min (Supplementary Figure 9a) correspond to M7BCGN2 ([M7BCGN2+Na]$^+$). (c) UPLC fraction (~16.0 min, Supplementary Figure 9a) containing M7BCGN2 was collected and treated with mannosidases, then analyzed by UPLC-MS. M7BCGN2 were digested with linkage-specific mannosidases, including: α1,2-mannosidase, which removes terminal α1,2 mannoses; α1,2-3-mannosidase, which removes terminal α1,2 mannoses and terminal α1,3 mannoses; α1,6-mannosidase, which removes terminal non-branched α1,6 mannoses. Digestion products and their deduced structure are depicted schematically: digestion of M7BCGN2 with α1,2-mannosidase produced M5A2BCGN2; with α1,2-3-mannosidase produced M3A3B2CGN2; while its digestion with α1,2-3-mannosidase and α1,6-mannosidase produced M1GN2.

Supplementary Tables

Supplementary Table 1. Summary of chemoenzymatic synthesized oligosaccharides.

Usual	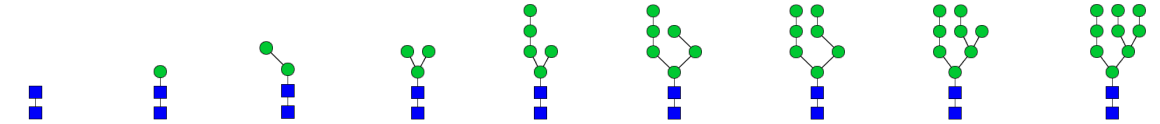								
	GN2	M1GN2	M2GN2	M3GN2	M5GN2	M6GN2	M7GN2	M8GN2	M9GN2
Unusual	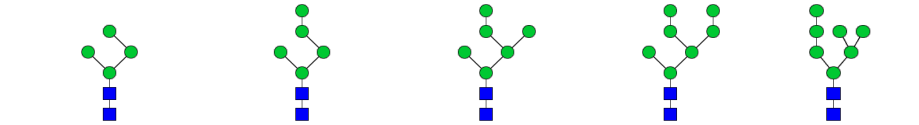								
	M4A2BC2GN2	M5A2C2GN2	M6A2CGN2	M7AGN2	M7BCGN2				
Digested	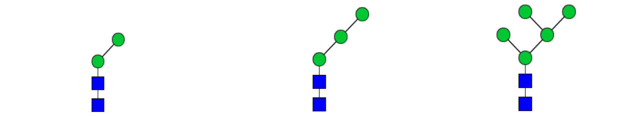								
	M2AGN2	M3A3B2CGN2	M5A2BCGN2						

Supplementary Table 2. Oligonucleotide primers used in this study.

Name	Sequences (5'-3')
Alg11-Fw	CGGAATTCGTGTATTCCAGCTTGAATCCGTT
Alg11-Rv	CCGCTCGAGTCAGCCCCTTTCCTCCTCTTCT
Dpm1-Fw	CGCGGATCCATGAGCATCGAATACTCTGTTA
Dpm1-Rv	CCGGAATTCTTAAAAGACCAAATGGTATA
Alg3-Fw	CGCGGATCCATGGAAGGTGAACAGTCTCCGC
Alg3-Rv	CCGCTCGAGTCAGTTGAGCTTTTTTTCCATAG
Alg9-Fw	CGGAATTCATGAATTGCAAGGCGGTAACCATTAG
Alg9-Rv	CCGCTCGAGTCAATTAGTAGTCTCAGTTG
Alg12-Fw	CGGAATTCATGCGTTGGTCTGTCCTTG
Alg12-Rv	CCGCTCGAGTCAATCAGTTTTTTCATC
Alg1-Fw	AAAACCATGGCGATCATCATATTTGTGCTGGG
Alg1-Rv	AAAAGGATCCTCAATGAATTAGCTTC
Rbs-Alg2-Fw	AAAAGAATTCTTTGTTTAACTTTAAGAAGGAG
Rbs-Alg2-rv	AAAAGAGCTCTTATATTTCTTCATAAGGG
Rbs-Alg11-Fw	AAAAGCGGCCGCTTTGTTTAACTTTAAGAAGGAGA
Rbs-Alg11-Rv	AAAACTCGAGTCAGCCCCTTTCCTC
Alg2-verify	AAAACCGATACTGTTGTGGTAAATTC

Supplementary Table 3. Plasmids used in this study.

Name	Descriptions	Parental plasmid and cloning sites
pET28-Mistic	T7 promoter; His6 tag at N-terminal	pET28; *Nde*I and *Nhe*I
pET28-Alg11ΔTM	T7 promoter; His6 tag at N-terminal	pET28; *EcoR*I and *Xho*I
pET28-Dpm1	T7 promoter; His6 tag at N-terminal	pET28; *BamH*I and *EcoR*I
pET28-Mistic-Alg3	T7 promoter; His6 and Mistic tag at N-terminal	pET28-Mistic; *BamH*I and *Xho*I
pET28-Mistic-Alg9	T7 promoter; His6 and Mistic tag at N-terminal	pET28-Mistic; *EcoR*I and *Xho*I
pET28-Alg12	T7 promoter; His6 tag at N-terminal	pET28; *EcoR*I and *Xho*I
pET28-Alg1ΔTM-Trx-Alg2-Alg11ΔTM	T7 promoter; His6 tag at N-terminal respectively	pET28; *Nco*I and *BamH*1, *Sac*I and *EcoR*I, *Not*I and *Xho*I

Supplementary Methods

NMR spectra were recorded in indicated solvents by using a Bruker Ultrashield 400 Plus or a Bruker Ascend 600 spectrometers. For the measurement in D2O, HDO signal (4.718 ppm at 30 °C) was used as a reference. *In situ* MALDI-TOF mass spectrometry was measured using a Bruker UltrafleXtreme. High resolution mass spectrometry (HRMS) was measured using a Waters SYNAPT G2-S or an Agilent 6220 TOF. Thin layer chromatography (TLC) analysis was performed on Merck 60 F254 silica-coated plates (Millipore, MA, USA) and visualized by sulfuric acid solution (5.0% in EtOH) with heating.

Supplementary Notes

Supplementary Note 1. Large scale enzymatic reactions.

To prepare milligram quantities of M5GN2 and M9GN2, reactions were performed in the following buffer: [14 mM MES/NaOH (pH 6.0), 4 mM potassium citrate, 10 mM $MgCl_2$, 10 mM $MnCl_2$, 0.05% NP-40, 1 M sucrose] in a total volume of 1 mL. Other conditions were as follows:

For production of M5GN2-PP-Phy: 0.2 mM GN2-PP-Phy, 2 mM GDP-Man and membrane fractions from *E. coli* that co-expressed Alg1ΔTM, Trx-Alg2 and Alg11ΔTM (50 μg/mL) were incubated at 30 °C for 12 h. Samples were then hydrolyzed with 20 mM hydrogen chloride. After 1 h incubation at 100 °C, the water-soluble glycan-containing fraction was desalted by solid-phase extraction using 1 mL Supelclean ENVI-Carb Slurry (Sigma-Aldrich, MO, USA) and lyophilized. The residue was further purified by reversed-phase chromatography (Sep-Pak C18, Waters) which was eluted by H_2O. The eluent was monitored by TLC with a 1-butanol/acetic acid/water (1:1:1, V/V/V) solvent, and the fractions containing M5GN2 (Rf = 0.40) were collected and lyophilized to give a white solid. One mg of GN2-PP-Phy resulted in 1 mg of the purified M5GN2, giving the recovery rate of 70%.

For production of M9GN2-PP-Phy: 0.3 mM GN2-PP-Phy, 3 mM GDP-Man and membrane fractions from *E. coli* that co-expressed Alg1ΔTM, Trx-Alg2 and Alg11ΔTM (60 μg/mL) were incubated at 30 °C for 12 h; then 3 mM Man-P-Phy, and membrane fractions from *E. coli* expressing [Mistic-Alg3 (60 mg/mL), Mistic-Alg9 (80 mg/mL) and Alg12 (80 mg/mL)] were added and incubated at 30 °C for 24 h.

Samples were hydrolyzed with 20 mM hydrogen chloride for 1 h at 100 °C. The water-soluble glycan-containing fraction was desalted by solid-phase extraction using 1 mL Supelclean ENVI-Carb Slurry (Sigma-Aldrich, MO, USA) and lyophilized. Glycans were further purified by reversed-phase chromatography (Sep-Pak C18, Waters) and eluted with H_2O. The eluent was monitored by TLC with a 1-butanol/acetic acid/water (1:1:1, V/V/V) solvent, and the fractions containing M9GN2 (Rf = 0.23) were collected and lyophilized to give a white solid. 0.6 mg of GN2-PP-Phy resulted in 0.5 mg of the purified M9GN2, yielding an overall recovery rate of 38%.

Supplementary Note 2. *In situ* MALDI-TOF MS analysis.

In situ MALDI-TOF MS analysis was performed before quenching the reaction by addition of hydrogen chloride. Typically, one droplet (50 µL) from the reaction was heated at 100 °C for 5 min then centrifuged to pellet the *E. coli* membrane. The supernatant was diluted 10 times with H_2O and subjected to mass analysis (Supplementary Figure 4), resulting in MALDI-TOF MS (negative): M5GN2-PP-Phy Anal. Calcd for $C_{66}H_{120}N_2O_{42}P_2$ (1674.68), found for m/z 1673.70 $[M-H]^-$; M9GN2-PP-Phy Anal. Calcd for $C_{90}H_{160}N_2O_{62}P_2$ (2322.89), found for m/z 2322.04 $[M-H]^-$.

In the M5GN2-PP-Phy reaction, the target compound was detected as the sole product (Supplementary Figure 4a). In the M9GN2-PP-Phy reaction, the target compound was observed as the main product but trace intermediates were also detected (Supplementary Figure 4b). These spectra demonstrated successful and efficient synthesis of both M5GN2-PP-Phy and M9GN2-PP-Phy.

Supplementary Note 3. Verification of the synthesized product.

The identity and purity of hydrolyzed M5GN2 and M9GN2 were verified by NMR (Supplementary Figure 5a-c) and HRMS. The ^{1}H NMR spectrum data of the final M9GN2 product was compared with the reported one[1], and chemical shifts of all protons were identical. In addition, through the two-dimensional NMR HSQC experiment (Supplementary Figure 5c), the other anomeric signal of one Man residue that was obscured by the water peak in ^{1}H NMR could be detected. By comparison with the previous reports[1-3] that assigned the anomeric protons of high-mannose type N-glycans (Supplementary Figure 5d), the anomeric signals of the M9CN2 synthesized in this work could be assigned as shown in Supplementary Figure 5c-d. From these data, it is concluded that the synthesized product corresponded to M9GN2.

Data were summarized below:

M5GN2:

^{1}H NMR (400 MHz, D_2O) δ 5.30 (s, 1H, H-1, Man), 5.26 (s, 1H, H-1, Man), 5.14 (brs, 1H, H-1, GlcNAc), 5.00 (s, 1H, H-1, Man), 4.87 (s, 1H, H-1, Man), 4.56 (d, 1H, $J_{1,2}$ = 9.5 Hz, H-1, GlcNAc), 4.31-3.41 (m, 42H, H-2,3,4,5,6 of Man and GlcNAc), 2.03 (s, 3H, GlcNAc), 1.99 (s, 3H, GlcNAc); HRMS Anal. Calcd for $C_{46}H_{77}N_2O_{36}$ $[M\text{-}H]^{-}$: 1233.4256, found: 1233.4291.

M9GN2:

^{1}H NMR (400 MHz, D_2O) δ 5.36 (s, 1H, H-1, Man), 5.29 (s, 1H, H-1, Man), 5.26 (s, 1H, H-1, Man), 5.14 (brs, 1H, H-1 (α), GlcNAc), 5.10 (s, 1H, H-1, Man), 5.01-5.00 (m, 3H, H-1, Man×3), 4.82 (s, 1H, H-1, Man), 4.55 (m, 1H, GlcNAc), 4.18-3.53 (m, 66H, H-2,3,4,5,6 of Man and GlcNAc), 2.02 (s, 3H, GlcNAc), 1.99 (s, 3H, GlcNAc); ^{13}C NMR (150 MHz, D_2O) δ 102.3 x 3 (C-1, Man x 3), 101.7 (C-1, GlcNAc), 100.8 (C-1, Man), 100.7 (C-1, Man), 100.5 (C-1, Man), 100.3 (C-1, Man), 99.8 (C-1, Man), 98.1 (C-1, Man), 94.9 (C-1 (β), GlcNAc), 90.4 (C-1 (α), GlcNAc), 22.2 (Ac, GlcNAc), 22.1 (Ac, GlcNAc); HRMS Anal. Calcd for $C_{70}H_{118}N_2O_{56}Na_2$ $[M+2Na]^{2+}$: 964.3116, found: 964.3100.

Supplementary References

1. Totani, K., Ihara, Y., Matsuo, I., Koshino, H. & Ito, Y. Synthetic substrates for an endoplasmic reticulum protein-folding sensor, UDP-glucose: glycoprotein glucosyltransferase. *Angewandte Chemie International Edition* **44**, 7950-7954 (2005).
2. Makimura, Y., Kiuchi, T., Izumi, M., Dedola, S., Ito, Y. & Kajihara, Y. Efficient synthesis of glycopeptide-α-thioesters with a high-mannose type oligosaccharide by means of tert -Boc-solid phase peptide synthesis. *Carbohydrate Research* **364**, 41-48 (2012).
3. Matsuo, I., Wada, M., Manabe, S., Yamaguchi, Y., Otake, K., Kato, K. & Ito, Y. Synthesis of monoglucosylated high-mannose-type dodecasaccharide, a putative ligand for molecular chaperone, calnexin, and calreticurin. *Journal of the American Chemical Society* **125**, 3402-3403 (2003).
