# Supplementary material for: Reconstitution of the lipid-linked oligosaccharide pathway for assembly of high-mannose N-glycans
Source: Nat Commun. 2019 Apr 18;10:1813. doi: 10.1038/s41467-019-09752-3 (PMC6472349; doi:10.1038/s41467-019-09752-3)
Supplement: Supplementary file 3 — Description of Additional Supplementary Files [file 41467_2019_9752_MOESM3_ESM.docx]

**Description of Supplementary Files**

**File Name:** Supplementary Data 1

**Description:** Sequencing data of the plasmids used in this study.
